# A Tumor-Agnostic, Topology-Informed Scoring Framework for Drug Repurposing: Application to CDK4/6 Inhibitor Resistance in HR^+^ Breast Cancer

**DOI:** 10.3390/biomedicines14030592

**Published:** 2026-03-06

**Authors:** Keyang Qian, Zijie Cai, Ruiquan Liu, Wang Yang, Jiayi Liu, Mengzi Wu, Mengdi Zhu, Linghan Wang, Huipei Gan, Zhuangqiu Yang, Xiaoting Jiang, Cailu Shen, Yong Mao, Qiang Liu

**Affiliations:** 1 Department of Oncology, The Affiliated Hospital of Jiangnan University, Wuxi 214062, China; qiankey759@163.com (K.Q.);; 2 Wuxi Medical College, Jiangnan University, No. 1800, Lihu Avenue, Wuxi 214122, China; 3 Guangdong Provincial Key Laboratory of Malignant Tumor Epigenetics and Gene Regulation, Guangdong-Hong Kong Joint Laboratory for RNA Medicine, Breast Tumor Center, Sun Yat-Sen Memorial Hospital, Sun Yat-Sen University, Guangzhou 510120, China; 4 Department of Plastic Surgery, Sun Yat-sen Memorial Hospital, Sun Yat-sen University, Guangzhou 510120, China; 5 Department of Endocrinology, Jiangnan University Medical Center, Affiliated Wuxi Clinical College of Nantong University, Wuxi No. 2 People’s Hospital, Wuxi 214002, China; 6 Department of Cancer Diagnosis and Treatment Center, Affiliated Hospital of Jiangnan University, No. 1000, Hefeng Road, Wuxi 214122, China

**Keywords:** drug repurposing, CDK4/6 inhibitor resistance, network topology, systems biology, *FGFR3*, sorafenib

## Abstract

**Background:** Therapeutic resistance to CDK4/6 inhibitors (CDK4/6i) remains a critical barrier in HR^+^ breast cancer. While network-based approaches offer a route to identify salvage therapies, existing methods often rely on inconsistent centrality metrics or retrospective public transcriptomes, lacking a unified framework to translate topology into pharmacological actionability. **Methods:** We developed the Topology-Integrated Hubness Score (TIHS), a quantitative framework that integrates five orthogonal network metrics into a unified hubness vector. To rigorously validate this framework and overcome the limitations of public bulk datasets, we combined cross-cohort statistical benchmarking with original RNA-sequencing data generated from a laboratory-derived palbociclib-resistant model (MCF7-PR). TIHS was applied to prioritize repurposing candidates by overlaying network hubness with drug–target affinity profiles. **Results:** Methodologically, TIHS demonstrated robust cross-dataset stability (cosine similarity ≥ 0.98) and statistically outperformed single-metric approaches in predicting drug sensitivity. In application, the framework identified sorafenib as a top-ranked candidate for reversing CDK4/6i resistance. Experimental validation confirmed these predictions: sorafenib significantly resensitized resistant cells (IC_50_ reduction from 6.57 μM to 1.15 μM), and molecular dynamics simulations supported stable binding to the TIHS-prioritized hub, *FGFR3*. Furthermore, functional assays involving siRNA-mediated knockdown validated that *FGFR3* is mechanistically required for the sorafenib resensitization phenotype. **Conclusions:** This study presents TIHS as a mechanism-agnostic, experimentally validated bridge between resistance-state transcriptomes and clinical decision-making. By coupling computational prioritization with in vitro functional verification, we demonstrate that targeting topology-defined hubs is a viable strategy for overcoming therapy resistance.

## 1. Introduction

CDK4/6i have transformed the treatment of hormone receptor–positive (HR^+^) breast cancer, but resistance is nearly inevitable and leaves patients with limited therapeutic options [1]. After progression on CDK4/6i–endocrine therapy, clinicians often face a time-critical decision window where multiple or unknown resistance mechanisms preclude a clear next step [2]. Existing salvage choices are empirical, and few frameworks can rapidly convert resistance-state data into actionable, evidence-based resensitization strategies. This clinical bottleneck underscores an urgent need for a reproducible, mechanism-agnostic route to identify new therapeutic opportunities after CDK4/6i failure [3].

Beyond CDK4/6 inhibitors, the therapeutic landscape of HR^+^ breast cancer has expanded to include antibody–drug conjugates (ADCs), PI3K/mTOR inhibitors, and novel drug-delivery strategies. Despite these advances, resistance across sequential treatment lines remains common, and optimal therapeutic sequencing continues to be debated. Recent work has highlighted both the translational challenges of nanomedicine-based strategies in breast cancer management and the evolving mechanisms of resistance associated with ADC therapies [4,5]. In parallel, emerging evidence suggests that regulatory RNA networks contribute to chemotherapy resistance and tumor progression, adding additional molecular complexity to treatment failure [6]. Collectively, these findings illustrate an increasingly multifactorial and adaptive resistance landscape that cannot be fully addressed by single-pathway or mutation-centric approaches.

Network-based approaches have therefore become central to modern drug discovery and repurposing in oncology, providing a systems-level view of cellular states and revealing key regulatory nodes—commonly termed “hubs.” These hubs are frequently considered high-priority therapeutic targets due to their centrality within disease-altered networks. Yet despite widespread application, the term ‘hubness’ remains conceptually ambiguous and inconsistently quantified. Studies often use varying thresholds or centrality metrics (e.g., degree, betweenness) to define ‘hub’ nodes, leading to low reproducibility and conflicting gene lists across platforms and datasets [7].

In the context of precision oncology, therapy resistance is increasingly recognized as a patient- and state-specific phenomenon rather than a uniform, generalizable process. Tumors that progress on the same therapy may exhibit distinct and concurrent resistance mechanisms, limiting the utility of pathway-centric or single-gene strategies and constraining the time available for exhaustive mechanistic dissection [8,9].

This lack of a unified, quantitative definition of hubness limits translational application. Tools such as CytoHubba [10], transcriptome-reversal platforms including the Connectivity Map (CMap) and LINCS [11,12,13], and mechanism-centric frameworks like OncoTarget/OncoTreat have each demonstrated value within their respective analytical scopes [14]. However, while these approaches often yield robust and reproducible computational results, the process by which high-dimensional transcriptomic or network-level outputs are translated into concrete therapeutic recommendations is frequently underexplained. This translational gap, rather than a lack of predictive accuracy, poses a practical challenge in time-sensitive resistant disease settings, where treatment decisions must be made before comprehensive mechanistic resolution is feasible.

**Figure 1:**
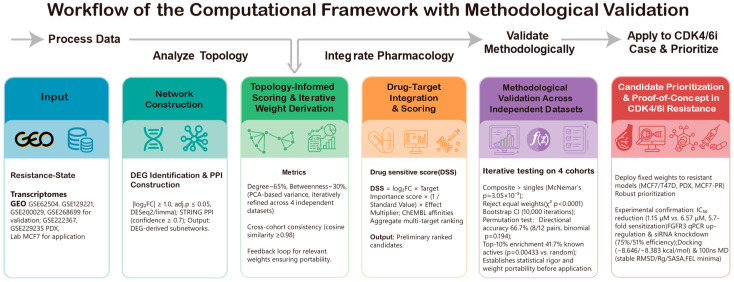
Overview of the topology-informed workflow used in this study. The framework comprises two major modules.

Conceptually (Figure 1), we sought to bridge this gap by formalizing “hubness” as a quantifiable, topology-weighted property and overlaying drug–target coverage to prioritize treatment options. We term this metric the Topology-Integrated Hubness Score (TIHS)—a mechanism-agnostic, tumor-type-independent approach that integrates five orthogonal protein–protein interaction (PPI)-network dimensions (degree, betweenness, eigenvector, MCC, EPC) into a unified score. It then overlays these network scores with curated drug–target interaction databases to assess which compounds best “cover” the most topologically critical nodes within a disease-shifted network. Unlike expression-signature-based methods, TIHS does not require prior knowledge of driver mutations or bypass pathways; by operating solely on the structure of the interactome and known pharmacological space, it provides a portable framework suitable for clinical timelines. By directly linking resistance-state transcriptomic reprogramming to drug–target space, this strategy aims to reduce intermediate analytical uncertainty. It thereby enables rapid and interpretable prioritization of candidate therapies within clinically relevant timelines.

This strategy addresses a key unmet need in real-world oncology, where drug resistance commonly arises from multiple, concurrent, and often unknown mechanisms. Tumor heterogeneity, clonal divergence, and incomplete knowledge of resistance biology frequently make pathway-specific interventions infeasible [15]. Instead of chasing one mechanism at a time, TIHS targets emergent network hubs that arise as tumors evolve, enabling mechanism-agnostic intervention and rapid, system-level prioritization of clinically available agents—an urgent requirement in post-resistance treatment decisions [16,17].

Resistance-associated network remodeling frequently involves receptor tyrosine kinase (RTK) signaling modules, which function as upstream coordinators of proliferative and adaptive programs. In receptor tyrosine kinase–driven resistance contexts, fibroblast growth factor receptor (*FGFR*) signaling has been implicated in tumor proliferation, survival, and adaptive escape mechanisms. Alterations in *FGFR* family members have been reported in subsets of breast cancer and therapy-resistant states, highlighting the broader relevance of RTK-mediated network remodeling during treatment adaptation [18,19,20].

In this study, we applied this framework to CDK4/6 inhibitor–resistant HR^+^ breast cancer, a setting of immediate clinical need, and evaluated its ability to generate experimentally testable, resistance-state–aware drug repurposing hypotheses.

## 2. Methods and Materials

### 2.1. Study Design and Analytical Workflow

In the framework training and methodological benchmarking module (upper panel), resistance-state transcriptomes from four independent GEO cohorts were preprocessed in a standardized manner, projected onto high-confidence STRING subnetworks, and used to compute five node-level topological metrics (degree, betweenness, eigenvector, maximal clique centrality [MCC], and edge percolated component [EPC]). These metrics were integrated into a composite hubness score, and dataset-specific weight vectors were derived via PCA-shift analysis. We then statistically benchmarked the composite score against individual metrics based on drug-ranking performance and averaged the per-dataset weight vectors to obtain a unified, validated weighting scheme.

In the resistance application module (lower panel), the locked TIHS weights were combined with curated drug–target interactions and potency measurements to prioritize repurposing candidates in CDK4/6 inhibitor–resistant breast cancer models, including public PDX datasets and an in-house palbociclib-resistant MCF7 transcriptome. Top-ranked candidates were subsequently evaluated through in vitro assays, molecular docking, molecular-dynamics simulations, and external clinical correlation. The following subsections detail data collection, preprocessing, scoring, and experimental validation.

### 2.2. Cohorts and Data Collection

#### 2.2.1. Framework Training and Validation Cohorts

To train and benchmark the TIHS, we first analyzed four GEO cohorts spanning distinct drugs, cancer types, and resistance contexts: GSE62504, GSE129221, GSE200029, and GSE268699. These datasets were used only to (i) construct resistance-associated PPI subnetworks, (ii) compare single-metric versus composite scoring performance, and (iii) derive dataset-specific weight vectors for the five topological metrics. The resulting vectors were then averaged to obtain a unified weighting scheme that was fixed a priori for all downstream resistance applications (see Section 3.2 and Supplementary Tables S3–S5).

For each cohort, we recorded sample size, platform, and group definitions (e.g., parental vs. resistant, pre- vs. post-treatment). Summary characteristics, including the number of samples per group and drug exposures, are provided in Supplementary Table S1. These cohorts were analyzed with identical thresholds and processing rules to ensure comparability across stages.

#### 2.2.2. Resistance Application Cohorts

We next applied the unified weighting scheme to three independent breast-cancer settings of CDK4/6 inhibitor resistance:

1. Primary test cohort (GSE222367)—paired parental MCF7/T47D lines and their palbociclib-resistant derivatives under clinically relevant palbociclib concentrations [21].
2. Internal validation cohort (GSE229235)—patient-derived xenograft (PDX) models generated under endocrine-sensitive versus palbociclib-resistant conditions.
3. External laboratory-derived model—an in-house palbociclib-resistant MCF7 line (MCF7-PR) for which we generated matched RNA-seq data [22].

All three cohorts were processed under identical thresholds and analysis rules to ensure comparability across stages. Group definitions and sample counts are summarized in Supplementary Table S1.

#### 2.2.3. External Clinical Cohort

To assess the clinical relevance of network-prioritized targets, we performed survival analyses using the TCGA breast cancer cohort (TCGA-BRCA). Overall survival data and gene-expression profiles were accessed via the KMplot platform (https://kmplot.com/analysis/, accessed on 10 December 2024), restricted to primary invasive breast carcinomas with available RNA-seq and follow-up information. Patients were stratified into high- and low-expression groups based on median *FGFR3* expression, and survival differences were evaluated as described in Section 2.9.

### 2.3. Data Preprocessing and Differential Expression Analysis

All GEO datasets were processed independently following platform-specific guidelines to minimize batch- and platform-related artifacts. Zero-expression features were removed before downstream analyses, and gene identifiers were standardized across platforms.

- GSE62504 (microarray): Raw intensities were log_2_-transformed and normalized according to the platform recommendations. Resistant versus sensitive contrasts were defined as in the original publication.
- GSE129221 (RNA-seq): Untreated parental and gefitinib-resistant PC9/PC9GR samples were extracted to construct baseline expression matrices for resistance contrasts.
- GSE200029 (RNA-seq): Vehicle-treated samples were selected to define parental versus tamoxifen-resistant groups in a manner consistent with the original design.
- GSE268699 (RNA-seq): We used the published log_2_ fold-changes contrasting fulvestrant- and palbociclib-treated resistant versus sensitive cells, maintaining consistency with the original preprocessing.
- GSE222367 and GSE229235 (RNA-seq): Raw counts or normalized expression matrices were imported, low-abundance genes filtered, and group labels assigned according to the original metadata.

For the laboratory-derived MCF7-PR model, RNA-seq data were processed analogously to the public RNA-seq cohorts (quality control, normalization, and log_2_ transformation) to preserve cross-dataset comparability.

#### 2.3.1. Definition of Resistance and Cohort-Specific Groupings

In GSE222367, palbociclib-resistant groups were consolidated at pharmacologically relevant concentrations of 3.6–4.8 μM, corresponding to clinically observed exposures (~3646–4425 nM). Operationally, palbociclib-resistant (PR) MCF7 and T47D lines were defined as those exhibiting ≥5-fold increases in IC_50_ relative to parental cells, consistent with prior pharmacologic profiling. In GSE229235, endocrine-resistant PDX models were classified as CDK4/6 inhibitor–sensitive, whereas PDX generated under palbociclib resistance were classified as resistant, following the original dataset’s methodology.

#### 2.3.2. Differential Gene Expression (DGE)

DGE analysis was conducted independently for each dataset to support both scoring validation and resistance-network construction. For the four framework training/benchmarking cohorts (GSE62504, GSE129221, GSE200029, GSE268699), we quantified resistance-associated transcriptional changes to test whether resistance patterns could be consistently captured across distinct biological systems. For the three resistance application cohorts (GSE222367, GSE229235, MCF7-PR), we focused on CDK4/6 inhibitor resistance and downstream network interpretation.

Unless otherwise stated, DGE was performed using DESeq2 (version 1.42.0) [23] with an adjusted *p*-value < 0.05 and |log_2_ fold change| ≥ 1.0. DEGs served as input for constructing high-confidence resistance networks in subsequent topological analyses.

### 2.4. PPI Network Construction and Topology-Integrated Hubness Scoring

To translate resistance-associated transcriptional signals into mechanistically plausible drug rankings, we integrated high-confidence PPI topology with target expression and drug potency. The working premise is that genes occupying structurally central positions in resistance networks are more likely to function as regulatory hubs under therapeutic pressure.

#### 2.4.1. PPI Network Construction

PPI networks were constructed in two stages. First, DEG-derived subnetworks were built for each of the four framework training/benchmarking cohorts (GSE62504, GSE129221, GSE200029, GSE268699), and the resulting networks were used to derive dataset-specific weight vectors for the five topological metrics. Second, the three resistance application cohorts (GSE222367, GSE229235, and MCF7-PR) were processed in the same way, but the averaged weights obtained from the training/benchmarking stage were fixed a priori and not recalculated.

For all cohorts, human PPI data were obtained from the STRING database (version 12.0). Interactions were restricted to edges with a combined score ≥ 0.7, corresponding to the high-confidence level defined by STRING [24], and this cutoff was applied uniformly across methodological-training and resistance-application stages. Lower thresholds (e.g., ≥0.4) increase edge density but introduce spurious links that destabilize centrality estimates, whereas stricter thresholds (>0.9) yield excessively sparse networks and can fragment biologically coherent modules. Applying a single, high-confidence threshold ensures robust topological inference and comparability across cohorts.

#### 2.4.2. Centrality Metrics, Normalization, and TIHS Definition

For each network, we computed five complementary centrality metrics for every node: degree centrality (DC), betweenness centrality (BC), eigenvector centrality (EC), maximal clique centrality (MCC), and edge percolated component (EPC). Each metric captures a distinct aspect of network influence:

- Degree identifies genes with the largest number of direct interactions and classic hub behavior [25].
- Betweenness identifies genes that bridge distant modules and may control information flow [26].
- Eigenvector emphasizes genes connected to other influential nodes [27].
- MCC highlights nodes embedded in densely interconnected sub-networks (cliques) [28].
- EPC reflects the robustness of nodes under edge perturbation and their contribution to network stability [10].

For each metric *j*, raw centrality values *X_ij_* were standardized via *Z*-score transformation:

$$Z_{ij}=\frac{X_{ij}-\mu_{j}}{\sigma_{j}}$$

where μ*_j_* and σ*_j_* are the mean and standard deviation of metric *j* across all nodes. To avoid negative values, we applied a shift transformation:

$$Z_{ij}^{\prime }=Z_{ij}+\left|min\left(Z_{j}\right)\right|+0.001$$

Weights for each metric (W*_j_*) were derived based on variance contributions and principal component loadings (PCA-shift) across all nodes. For a given dataset, the weight for metric j was defined as:

$$W_{j}=\frac{\sigma_{j}}{\sum_{k=1}^{n}\sigma_{k}}$$

PCA was implemented in R software (version 4.3.2, R Foundation for Statistical Computing, Vienna, Austria) using the prcomp function from the base stats package, and weight vectors were derived from component loadings. The final weights applied in all resistance-model analyses were the averaged vector obtained from the four training/benchmarking datasets, providing a statistically validated weighting scheme rather than an arbitrary equal-weight assumption.

The TIHS for each node *i* was then computed as:

$$\mathrm{TI}{HS}_{i}=\sum_{j=1}^{n}W_{j}\cdot Z_{ij}^{\prime }$$

where *n* denotes the total number of metrics. TIHS thus integrates multiple topological dimensions into a single continuous measure of hubness, enabling consistent and biologically grounded prioritization of network hubs across datasets.

#### 2.4.3. Cross-Dataset Stability of Weight Vectors

To evaluate the stability and cross-model consistency of derived topological weights, we computed cosine similarity between each dataset-specific weight vector *w_i_* and the unified mean vector *w*_unified_:

$$\mathrm{cosine}\text{\_}\mathrm{sim}\left(w_{i},w_{\mathrm{unified}}\right)=\frac{w_{i}\cdot w_{\mathrm{unified}}}{\left|w_{i}|\;|w_{\mathrm{unified}}\right|}$$

Weight vectors were normalized to unit length before computation. Calculations were performed in R (v4.3.2) using custom functions and confirmed with the *lsa* package. Results were visualized in polar coordinates using *ggplot2* and *ggrepel*, with a dashed ring at 0.98 indicating the stability threshold (Figure 4d). High cosine similarity (≥0.98 for all datasets) indicated that the relative ranking of the five topological metrics was preserved across independent models, supporting the robustness and generalizability of the weighted composite framework.

### 2.5. CDK4/6i Resistance Heterogeneity Assessment

To quantify transcriptional variability within CDK4/6 inhibitor resistance models, gene-level Log_2_FC values (resistant vs. sensitive/parental) were extracted from four CDK4/6i-resistant datasets. Only intersected genes shared across all four models were retained to construct a genes × models resistance-shift matrix.

Principal component analysis (PCA) was performed on model-level shift vectors using centered data without scaling (R function prcomp, center = TRUE, scale = FALSE). Pairwise similarity between models was quantified using Pearson correlation computed across genes. Hierarchical clustering was conducted using complete linkage on 1—correlation distance.

To evaluate the robustness of PCA-based separation under high dimensionality, gene-level bootstrap resampling was performed (500 iterations), in which genes were sampled with replacement and PCA recomputed for each iteration. Model coordinate distributions were summarized in principal component space.

### 2.6. Drug–Target Database and TIHS-Based Drug Prioritization

#### 2.6.1. Construction of the Anticancer ChEMBL Subset

To identify candidate drugs capable of targeting resistance-state hubs, we constructed a specialized anticancer subset of the ChEMBL 34 database [29]. Antineoplastic agents were selected based on the Anatomical Therapeutic Chemical (ATC) “L” classification, and corresponding molregno identifiers were extracted [30]. For each drug, we retrieved human targets, UniProt IDs, standard activity values (e.g., IC_50_, EC_50_), units, and mechanism of action (inhibitor, antagonist, agonist, etc.) from *the activities* and *drug_mechanism* tables.

All activity values were converted to nM to allow cross-drug comparison. This yielded a curated drug–target table linking each anticancer compound to one or more human protein targets with standardized potency annotations, forming the basis for TIHS-based sensitivity scoring.

#### 2.6.2. Drug-Target Matching and Sensitivity Score Calculation

Topological features (TIHS) were derived from the PPI networks as described above and linked to drug–target data to compute a drug sensitivity score for each dataset. For every drug–target pair, three components were integrated:

1. The log_2_ fold change in the target gene between resistant and sensitive samples.
2. The standardized activity value of the drug (e.g., IC_50_/EC_50_, in nM).
3. The target’s TIHS value reflecting its network importance.

For each target-metric combination, the shifted *Z*-score and metric weight were used to build a composite contribution. The total score for a given drug was defined as:

$$\mathrm{Total}\;\mathrm{Score}=\sum_{j}\left(\frac{\log2\;FoldChange}{\mathrm{standard}\mathrm{value}}\cdot {\mathrm{Shifted}}_{j}\cdot 100\cdot w_{j}\right)$$

where “Standard Activity” is the activity value in nM, Shifted*_j_* is the normalized and shifted *Z*-score for metric *j*, and *w_j_* is the corresponding weight.

To account for mechanism of action, we applied an effect multiplier:

- +1 for inhibitory or suppressive drugs.
−1 for agonists or activators.

The final drug sensitivity score was:

*Drug sensitive score* = *Total Score* × *Effect Multiplier*

Drugs with positive scores were interpreted as candidates predicted to be more effective in the resistant state, whereas negative scores indicated predicted resistance.

#### 2.6.3. Multi-Tiered False-Positive Control Pipeline

To mitigate false positives—a common challenge in network-based drug repurposing—and to ensure the TIHS framework is generalizable to single-dataset clinical applications beyond the rigorous multi-cohort intersection used in our initial proof-of-concept, we established a multi-tiered filtering strategy for candidate prioritization:

1. Topological and Ranking Threshold: Based on our empirical validation demonstrating that known active drugs are significantly enriched in the top decile (Figure 3f), candidates must achieve a positive total matching score and rank within the top 10% of all evaluated agents.
2. Target Multiplicity Threshold: To buffer against bypass-track resistance and network redundancy, candidates must map to ≥2 topological targets. Single-target agents are thereby deprioritized in favor of multi-kinase or multi-node inhibitors (e.g., sorafenib).
3. Biological and Pharmacokinetic Filters: Topologically identified targets must demonstrate detectable endogenous absolute expression (e.g., excluding targets with negligible mRNA abundance such as *FLT3*). Finally, the predicted or validated in vitro IC_50_ of the candidate must fall within the clinically achievable maximum plasma concentration (*C_max_*) to ensure translational feasibility.

#### 2.6.4. Summary of Drug–Target Matching and Cross-Model Benchmarking

For each dataset, we computed sensitivity scores for all candidate drugs and summarized:

- Total score (cumulative weighted sensitivity score across matched targets);
- Number of matched targets per drug;
- Average standard activity value across targets;
- List of matched gene names and target details.

We then compiled a set of 12 literature-supported drug–phenotype pairs across cohorts and evaluated whether TIHS correctly predicted (i) the therapeutic direction (sensitive vs. resistant) and (ii) a top-decile ranking (top 10% of candidates). The observed hit rate was compared with the 10% random expectation using a one-sided exact binomial test, as detailed in Section 2.10.

### 2.7. Cell Lines, Culture Conditions, and In Vitro Functional Assays

#### 2.7.1. Cell Lines and Generation of Palbociclib-Resistant MCF7-PR

Parental MCF7 cells (MCF7-P) were obtained from an authenticated commercial source and cultured in standard DMEM supplemented with 10% fetal bovine serum, penicillin/streptomycin, and 5% CO_2_ at 37 °C.

Palbociclib-resistant MCF7 cells (MCF7-PR) were generated by long-term stepwise dose escalation of palbociclib. Cells were initially exposed to 100 nM palbociclib, and the concentration was gradually increased over approximately six months until a final maintenance concentration of 6 μM was reached. Resistant populations were continuously cultured in medium containing 6 μM palbociclib for maintenance. Prior to experimental assays, resistant cells were refreshed in palbociclib-free medium for 48 h to eliminate acute drug effects. Stable resistance (≥5-fold IC_50_ shift compared with parental cells) was confirmed by repeated dose–response assays before RNA-seq and functional experiments.

#### 2.7.2. Sorafenib Dose–Response Assays (MTT)

Sorafenib sensitivity was assessed using MTT assays in parental MCF7-P and resistant MCF7-PR cells. Cells were seeded at 4000–5000 cells per well in 96-well plates and incubated overnight. Sorafenib (Selleck, Houston, TX, USA, S7397) was dissolved in DMSO and applied at eight concentrations prepared by 3-fold serial dilutions (60 μM to ~0.01 μM), with six replicate wells per concentration. After 72 h of exposure, cell viability was measured using MTT (Asegene Biotec Co., Ltd., Guangzhou, China, #43200), and absorbance was recorded at 570 nm. Log-transformed dose–response data were fitted using nonlinear regression in GraphPad Prism (version 8.0.2, GraphPad Software, San Diego, CA, USA) to estimate IC_50_ values for each condition.

#### 2.7.3. RT-qPCR and RNA Interference

Total RNA was extracted using the GOONIE Rapid RNA Extraction Kit (BioTeke, Guangzhou, China), and cDNA was synthesized with HiScript III All-in-one RT SuperMix (Vazyme, Nanjing, China). RT-qPCR was performed with ChamQ Universal SYBR qPCR Master Mix (Vazyme, China) on an ABI QuantStudio DX instrument, with three technical replicates per sample. Primer sequences for *FGFR3*, *FLT3*, and reference genes were designed using the Deep BioGroup platform and synthesized by IGE Biotech (Guangzhou, China). Specificity was confirmed by melting-curve analysis (primer sequences in Supplementary Table S9).

For RNA interference, MCF7-PR cells were seeded in 6-well plates (1 × 10^6^ cells/well) and transfected at 50–70% confluence. For each well, 100 pmol of *FGFR3*-targeting or negative-control siRNA (GenePharma, Suzhou, China) was mixed with 7.5 μL Lipofectamine 3000 (Invitrogen, Carlsbad, CA, USA) in Opti-MEM (Gibco, Waltham, MA, USA), incubated for 15 min at room temperature, and added to cells. The medium was replaced with complete growth medium after 6–8 h. Knockdown efficiency was assessed at the mRNA level by RT-qPCR and at the protein level by Western blotting.

#### 2.7.4. Western Blot Analysis

For Western blotting, cells were lysed in RIPA buffer supplemented with protease and phosphatase inhibitors. Equal amounts of protein were separated by SDS–PAGE and transferred to PVDF membranes. Membranes were blocked with 5% non-fat milk and incubated with primary antibodies against *FGFR3* and GAPDH (loading control), followed by appropriate HRP-conjugated secondary antibodies. Signals were detected using chemiluminescence and quantified by densitometry. *FGFR3* expression was normalized to GAPDH and expressed relative to the non-targeting siRNA control.

### 2.8. Molecular Docking and Molecular Dynamics Simulation

Molecular docking was performed using AutoDock Vina [31] (v1.1.2) to evaluate sorafenib binding to *FGFR3* and *FLT3*. Crystal structures of *FGFR3* (PDB ID: 9CD7, 2.7 Å) and *FLT3* (PDB ID: 6JQR, 2.4 Å) were obtained from the RCSB PDB [32,33]. Co-crystallized ligands and water molecules were removed, hydrogen atoms added, and structures converted to PDBQT using AutoDock Tools and PyMOL Molecular Graphics System (version 3.1.3, Schrödinger, LLC, New York, NY, USA). The 3D structure of sorafenib was retrieved from PubChem and converted to PDBQT via Open Babel (version 3.1.1)dd with geometry optimization [34].

Docking grids (30 Å × 30 Å × 30 Å, 1.0 Å spacing) were centered on the ATP-binding site of each kinase based on co-crystallized ligands. Exhaustiveness was set to 10, and 20 binding modes were generated. Poses were ranked by predicted binding free energy (ΔG, kcal/mol), and representative low-energy complexes were selected for MD simulation.

MD simulations were carried out with GROMACS (version 2022.3) [35] using the CHARMM36 [36] force field for proteins and CGenFF [37] parameters for sorafenib. Complexes were solvated in a TIP3P water box with a 1 nm buffer, neutralized with counter ions, and equilibrated at 310 K and 1 bar [38]. Production runs were 100 ns with a 2-fs timestep. RMSD, RMSF, Rg, SASA, hydrogen bonds, and free-energy landscapes (FEL) were analyzed using standard GROMACS tools. Binding free energies and per-residue contributions were estimated using MM/PBSA [39] on snapshots from the last 20 ns.

### 2.9. Survival Analysis

The prognostic impact of *FGFR3* and *FLT3* expression was evaluated in TCGA-BRCA using KMplot (https://kmplot.com/analysis/, accessed on 10 December 2024) [40,41]. Patients were dichotomized into high- and low-expression groups using the median expression level automatically calculated by the KMplot platform for each gene. Overall survival was compared between groups with Kaplan–Meier analysis, and log-rank tests were used to assess statistical significance. Hazard ratios (HRs) and 95% confidence intervals (CIs) were reported, with *p* < 0.05 considered significant.

### 2.10. Statistical Analysis

Unless otherwise specified, all statistical analyses were performed in R software (version 4.3.2, R Foundation for Statistical Computing, Vienna, Austria) or GraphPad Prism (version 8.0.2, GraphPad Software, San Diego, CA, USA).

- Comparison of composite versus single metrics: For each dataset–cell line–drug pair, predictions were encoded as correct/incorrect, and 2 × 2 contingency tables were constructed. McNemar’s test was applied to discordant pairs (n_10_ vs. n_01_) to assess whether the composite score achieved significantly higher paired accuracy than any single centrality metric.
- Testing the equal-weight assumption and weight stability: χ^2^ goodness-of-fit tests (df = 4) were used to compare dataset-specific weight distributions against a 0.2:0.2:0.2:0.2:0.2 null. Cross-dataset homogeneity was assessed with χ^2^ tests, and concordance was quantified by cosine similarity and Pearson correlation with the averaged vector.
- Directional prediction validation: For literature-supported drug–phenotype pairs, directional accuracy (sensitive vs. resistant) was summarized as a proportion. Bootstrap resampling (10,000 iterations) was used to estimate 95% confidence intervals, and permutation tests (10,000 label shuffles) were used to assess whether observed accuracy exceeded random expectation.
- Ranking-based validation: For each validated drug, the direct *p*-value was defined as rank_obs/N_cand. Permutation-based *p*-values were obtained by comparing rank_obs/N_cand to 10,000 uniform random values in [0, 1], representing a null of random ranking.
- Cross-model enrichment: The observed fraction of validated drugs falling within the top 10% of candidates was compared to the 10% null expectation using a one-sided exact binomial test.
- In vitro assays: RT-qPCR data were analyzed using two-tailed *t*-tests; MTT dose–response curves were fitted by nonlinear regression, and IC_50_ values were compared using extra sum-of-squares F-tests. Western blot densitometry was analyzed with *t*-tests or ANOVA where appropriate.
- Survival analysis: Kaplan–Meier curves were compared by log-rank tests, with HRs and 95% CIs derived from Cox proportional-hazards models.

*p*-values < 0.05 were considered statistically significant unless otherwise noted.

## 3. Results

**Figure 2:**
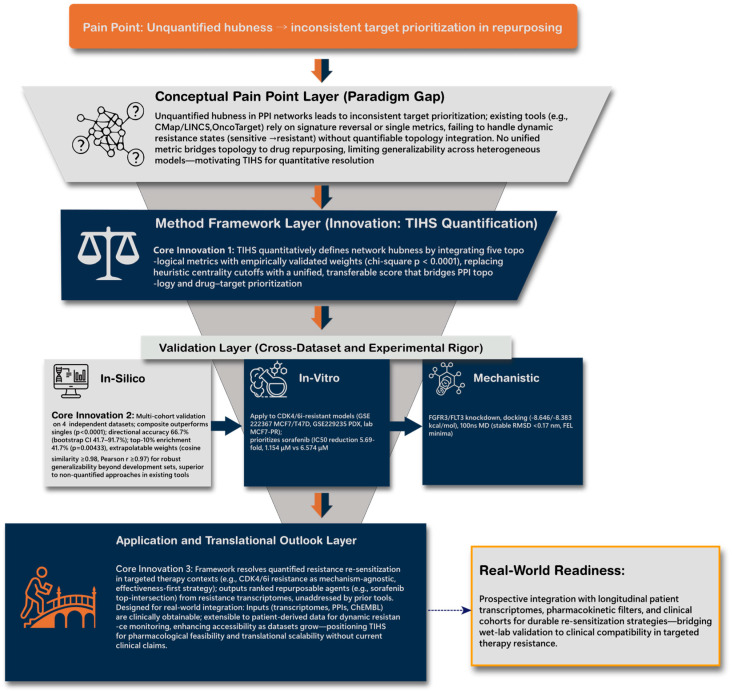
Quantitative topology-based framework resolving target prioritization in CDK4/6 inhibitor–resistant breast cancer.

We first implemented a four-layer topology-informed framework to transform qualitative PPI-network structure into a unified, quantitative measure of hubness, termed the Topology-Integrated Hubness Score (TIHS; Figure 2). In the Conceptual Layer, TIHS defines hubness as a weighted integration of five centrality metrics (degree, betweenness, eigenvector, MCC, EPC), and in the Validation and Application Layers we assessed whether this composite score improves drug-sensitivity prediction across independent resistance datasets and in CDK4/6 inhibitor–resistant HR^+^ breast-cancer models.

Unquantified hubness in traditional PPI analyses causes inconsistent target ranking across repurposing datasets (top). The proposed TIHS redefines hub importance by integrating five validated network metrics into a unified, quantitative score that links topology to pharmacologic actionability (middle). Cross-dataset and experimental validations in CDK4/6i-resistant HR^+^ models (MCF7/T47D, PDX) confirmed reproducible target prioritization and sorafenib–*FGFR3*–mediated resensitization (bottom). This multi-layer hierarchy bridges systems computation to clinically actionable retreatment strategies.

### 3.1. Validation of the Topology-Integrated Scoring Framework

All validation analyses were performed on DEG-derived, high-confidence PPI networks constructed from multiple resistance-associated transcriptomic datasets across distinct drug classes (see Section 2). DEGs were defined as |log_2_ fold change| ≥ 1.0 with adjusted *p* ≤ 0.05, and STRING [42] interactions were restricted to links with confidence ≥ 0.7. To test the methodological validity of the framework, we first asked whether integrating multiple topological attributes improved predictive performance relative to any single attribute and then examined how weight distributions influenced the composite (see Supplementary Table S1).

#### 3.1.1. Equal-Weighted Composite Versus Single Metrics

**Figure 3:**
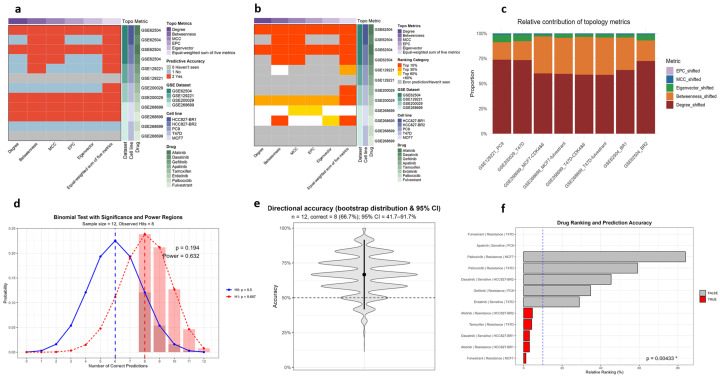
Statistical validation of the TIHS framework across multiple resistance datasets. (**a**) Paired accuracy advantage of the equal-weighted composite over any single topology metric (McNemar, one-sided; n_10_ = 15, n_01_ = 0; *p *= 3.05 × 10^−5^). (**b**) Enrichment among top ranks: validated drugs more often fall within the top 10% and 30% under the composite vs. single metrics (McNemar, one-sided; Top10 n_10_ = 20, n_01_ = 0; *p *= 9.54 × 10^−7^; Top30 n_10_ = 20, n_01_ = 0; *p* = 9.54 × 10^−7^). (**c**) Weighting evidence: the equal-weight assumption is rejected and cross-dataset homogeneity is not supported, while per-dataset weight vectors remain highly concordant with the across-dataset mean. (**d**) Directionality: the weighted-sum score predicts sensitivity versus resistance in 8/12 curated cases (66.7%), with bootstrap 95% confidence intervals and permutation-based *p*-values described in Methods. In the binomial test illustration, the darker red shaded region denotes the rejection (significance) region defined by the critical threshold, whereas the lighter red bar highlights the observed number of correct predictions. (**e**) Violin plot of bootstrap resampling (10,000 iterations) shows the distribution of directional accuracy; the central dot indicates the observed value (8/12), the bar the 95% bootstrap confidence interval, and the dashed line the 50% chance level. (**f**) Prioritization: 5/12 (41.7%) correctly predicted drugs’ rank within the top 10% (with permutation-based enrichment *p*-value), well above the 10% random expectation. See Supplementary Tables S1–S5 for full statistics and per-dataset details. * indicates statistical significance (*p* < 0.05).

Across all validation sets, the equal-weighted composite consistently outperformed any single metric. McNemar’s test confirmed significantly higher overall prediction accuracy (n_10_ = 15 vs. n_01_ = 0; *p *= 3.05 ×10^−5^), with similar advantages in capturing validated drugs within the top 10% (n_10_ = 20 vs. n_01_ = 0; *p *= 9.54 × 10^−7^) and top 30% (n_10_ = 20 vs. n_01_ = 0; *p *= 9.54 × 10^−7^) of candidates (Figure 3a,b; Table S2). Degree, EPC, and eigenvector centralities showed significant gains, while MCC and betweenness trended positively but did not reach significance. These results demonstrate that integrating multiple attributes is statistically superior to reliance on any single metric.

#### 3.1.2. Equal-Weight Assumption and Weighting Stability

We next tested whether equal weighting of the five topological attributes was justified. χ^2^ goodness-of-fit tests consistently rejected the equal-weight assumption across all datasets (all *p* < 0.0001; Table S4). Cross-dataset homogeneity testing further revealed significant differences in weight proportions (χ^2^ = 31,445, df = 28, *p* < 2.2 × 10^−16^), with degree and betweenness dominating and eigenvector, MCC, and EPC contributing less (Table S3). Despite this heterogeneity, dataset-specific weight vectors were highly concordant with the across-dataset mean (cosine similarity ≥ 0.98; Pearson r ≥ 0.97; Table S5). Detailed χ^2^ outputs and cross-dataset similarity matrices are shown in Supplementary Tables S4 and S5. Thus, while equal weighting is statistically inappropriate, the stability of weight patterns across datasets supports the use of robust weighted integration schemes (Figure 3c). Accordingly, we adopted the across-dataset mean weight vector as the unified weighting scheme for subsequent analyses.

### 3.2. Weighted Integration Confirms Predictive Validity

Having established that equal weighting improves upon any single metric but does not reflect the true weight distribution, we next evaluated the predictive validity of the weighted TIHS. Among 12 curated drug–dataset pairs with prior biological evidence, the model correctly classified 8, yielding a directional accuracy of 66.7% (binomial *p* = 0.194; Table 1). Although this test did not reach conventional significance, likely reflecting the limited number of curated pairs, rank-based enrichment was more informative: 5/12 (41.7%) correctly predicted drugs simultaneously ranked within the top 10% of candidates—well above the 10% random expectation (permutation *p* = 0.00433; Figure 3d–f). Together, these results establish the weighted topology-integrated framework as a statistically grounded and informative predictor of drug sensitivity, and we therefore locked the averaged weight vector derived from the four validation sets for downstream applications in resistant models.

### 3.3. Transcriptomic and Functional Characterization of CDK4/6 Inhibitor Resistance in Breast Cancer

We then profiled resistance-associated transcriptomic changes across the primary test dataset (GSE222367), the internal validation PDX cohort (GSE229235), and the laboratory-derived MCF7-PR model to contextualize downstream network analyses and drug prioritization (Section 2.1 and Section 2.2).

#### 3.3.1. Results of Differential Gene Analysis

Following the statistical validation of TIHS (Figure 3), we performed differential expression analysis across three independent models of CDK4/6 inhibitor resistance—GSE222367 cell lines (MCF7, T47D), the PDX cohort (GSE229235), and the lab-derived MCF7-PR model—to construct resistance-related PPI networks. In the primary test dataset (GSE222367), 2664 genes were downregulated and 2516 upregulated in palbociclib-resistant MCF7 cells, while T47D cells exhibited 3565 downregulated and 4045 upregulated genes. In the internal validation PDX cohort, 3307 genes were downregulated and 5343 were upregulated in resistant samples. For the external lab-derived resistant MCF7 model, 2084 genes were downregulated and 1455 were upregulated (Supplementary Figure S1). These gene expression changes provided the basis for constructing high-confidence resistance networks in subsequent topological analyses.

Analysis of canonical resistance genes across the three models revealed a clear pattern of heterogeneity. RB1 downregulation was observed only in the MCF7 subset of GSE222367 and in the lab-derived resistant MCF7 cell line, but not in the PDX model. Conversely, *FGFR1* amplification was detected exclusively in the PDX model and in the lab-derived MCF7 resistant cells but was absent in the GSE222367 cell lines. These findings illustrate the variable presentation of established resistance mechanisms among different CDK4/6 inhibitor–resistant models.

#### 3.3.2. Topological Weight Derivation and Cross-Dataset Stability

**Figure 4:**
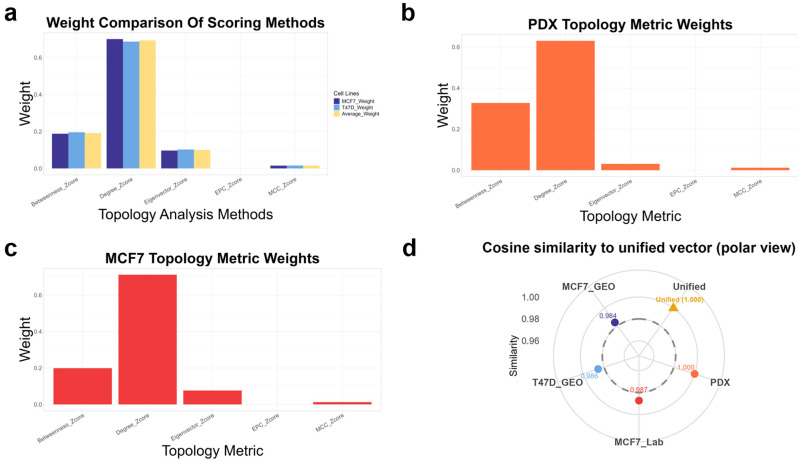
Statistical validation of dataset-specific topological weights and cross-model stability. (**a**–**c**) Relative weights of five topology metrics (degree, betweenness, eigenvector, MCC, EPC) derived from PCA-shift analysis in three representative contexts: (**a**) MCF7/T47D average, (**b**) PDX model, and (**c**) laboratory MCF7-palbociclib-resistant model. (**d**) Polar plot showing cosine similarity of each dataset’s weight vector to the unified mean (cosine ≥ 0.98), indicating stable ranking patterns across models.

PPI networks were constructed for the four datasets using the above DEG filters and STRING interactions. For the MCF7 and T47D cell lines in the test set, 3959 and 5171 protein targets were obtained, respectively, with 8044 and 13,613 high-confidence interactions retained after filtering. For the internal PDX model, 5836 targets and 23,358 high-confidence edges were identified. These PPI networks were then subjected to topological quantification using five centrality metrics (degree, betweenness, eigenvector, MCC, EPC), and dataset-specific weights were derived through PCA-shift analysis (Figure 4a–c). Across all models, degree and betweenness consistently dominated the composite score, whereas eigenvector, MCC, and EPC contributed minimally, confirming a non-equal yet robust weighting pattern.

To evaluate the cross-dataset stability of these weight distributions, we calculated the cosine similarity between each dataset’s weight vector and the unified mean vector (Figure 4d). All datasets showed high similarity (cosine ≥ 0.98), indicating that although the absolute proportions differed, the relative ranking of metric importance was conserved. Consequently, the weighted network-importance score simultaneously captures dataset-specific nuances and strong cross-model generalizability, forming a stable structural basis for subsequent analyses.

#### 3.3.3. Transcriptomic Heterogeneity Within CDK4/6i Resistance Models

While topology-derived weights exhibited strong cross-model concordance, the extent of transcriptional similarity among CDK4/6i-resistant models remained unclear. To characterize transcriptional variability within CDK4/6 inhibitor resistance contexts, we constructed gene-level Log_2_FC resistance shift profiles for each dataset and retained intersected genes across all four CDK4/6i-resistant models to ensure comparability. Principal component analysis showed clear separation among models in PC space (Supplementary Figure S2a).

Consistently, pairwise Pearson correlations of model-level resistance shift vectors ranged from −0.114 to 0.463 (Supplementary Figure S2c). Only MCF7_GEO and T47D_GEO exhibited moderate similarity (r = 0.463), whereas other comparisons showed near-zero or weak negative correlations, indicating substantial divergence across CDK4/6i resistance models.

To evaluate the robustness of the PCA separation under this high-dimensional setting, gene-level bootstrap resampling was performed (n = 500 iterations). Bootstrap distributions demonstrated stable positioning of each model in principal component space (Supplementary Figure S2b).

#### 3.3.4. Drug-Target Mapping and Validation Results

**Figure 5:**
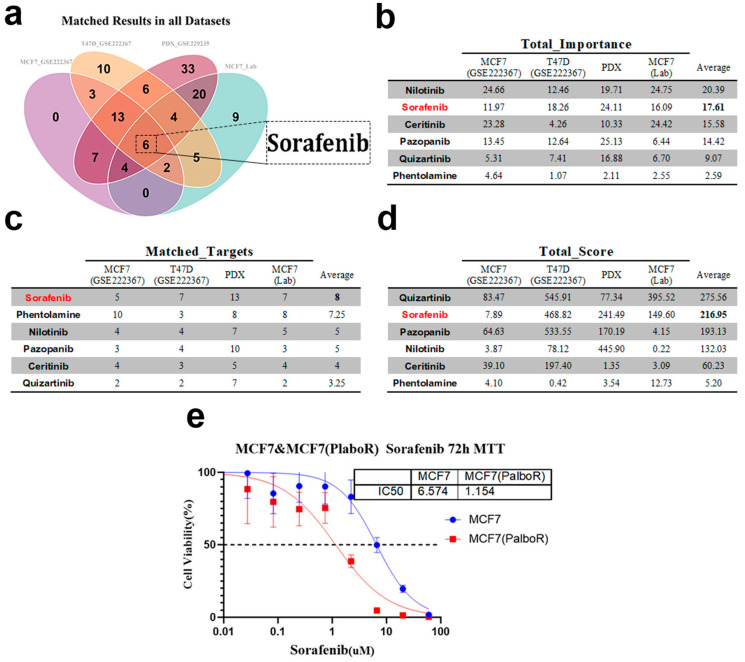
Application of the TIHS-based scoring to identify candidate therapeutic agents. (**a**) Venn diagram showing the intersection of sensitive drugs identified across the datasets (**b**) Total importance values for six drugs across various datasets, highlighting their relevance to the study. Sorafenib emerges as a leading candidate with high importance scores in all datasets. (**c**) Number of matched targets for each drug, showing the interaction strength between drug candidates and their corresponding targets across the datasets. (**d**) Total scores for each drug, reflecting their performance across the test, internal, and external validation sets. Sorafenib shows a strong overall performance, ranking among the top drugs. (**e**) MTT assay results for Sorafenib treatment on MCF7 and MCF7-PR cells. IC_50_ values are provided to demonstrate Sorafenib’s dose–response relationship, with a significant decrease in IC_50_ for resistant cells compared with parental MCF7 cells.

Building upon the topology-weighted resistance networks described above, we next translated these network representations into actionable drug candidates. We matched a curated anticancer drug–target affinity database from ChEMBL to each dataset to obtain drug–target matching results. A negative total matching score suggested potential drug resistance, while a positive score indicated possible drug sensitivity. Drug candidates were ranked based on a composite of TIHS-based target importance (Topology-Integrated Hubness Score), the number of matched targets, and drug sensitivity scores (DSS). Drugs with a total matching score greater than zero were selected for further evaluation, and those with more than one matched target were prioritized. In total, 34, 48, 93, and 49 sensitive drugs were identified across the four datasets, respectively (Table S6). The intersection of these drug lists identified six agents that were predicted to be sensitive in all datasets (Figure 5a).

Notably, sorafenib consistently ranked among the top candidates across multiple datasets, with high importance, target coverage, and composite sensitivity scores (Figure 5b–d). External validation confirmed this prediction: dose–response assays showed that sorafenib reduced the IC_50_ from 6.574 μM in parental MCF7 cells to 1.154 μM in resistant MCF7-PR cells (R^2^ > 0.7), representing a 5.69-fold difference in sensitivity (Figure 5e). This result aligns with the computational ranking and highlights sorafenib as a potent therapeutic option for overcoming CDK4/6 inhibitor resistance. Importantly, both *FGFR3* and *FLT3* emerged as recurrent sorafenib-binding candidates in our framework; however, *FLT3* expression was low in resistant MCF7 cells, and its effective concentrations exceeded clinically achievable plasma levels (Cmax ≈ 2–3 μM), limiting its translational relevance. In contrast, *FGFR3* was consistently upregulated and centrally positioned within resistance networks, making it a more tractable and biologically plausible candidate for downstream validation. Based on these considerations, subsequent experiments focused on *FGFR3* as a potential driver of sorafenib sensitivity.

In addition to sorafenib, five other agents—nilotinib, quizartinib, pazopanib, ceritinib, and phentolamine—were recurrently prioritized across datasets, forming a set of six top candidate drugs by intersection analysis. However, in vitro IC_50_ validation revealed that nilotinib showed no marked differences between parental and resistant cells, and quizartinib, although showing a modest shift, exhibited absolute IC_50_ values substantially above clinically achievable concentrations and a smaller fold change than sorafenib. Pazopanib, ceritinib, and phentolamine also failed to exert significant inhibitory effects in resistant models (Figure S3). These results collectively reinforced sorafenib as the most robust candidate for detailed functional validation in this study.

#### 3.3.5. Molecular Docking

**Figure 6:**
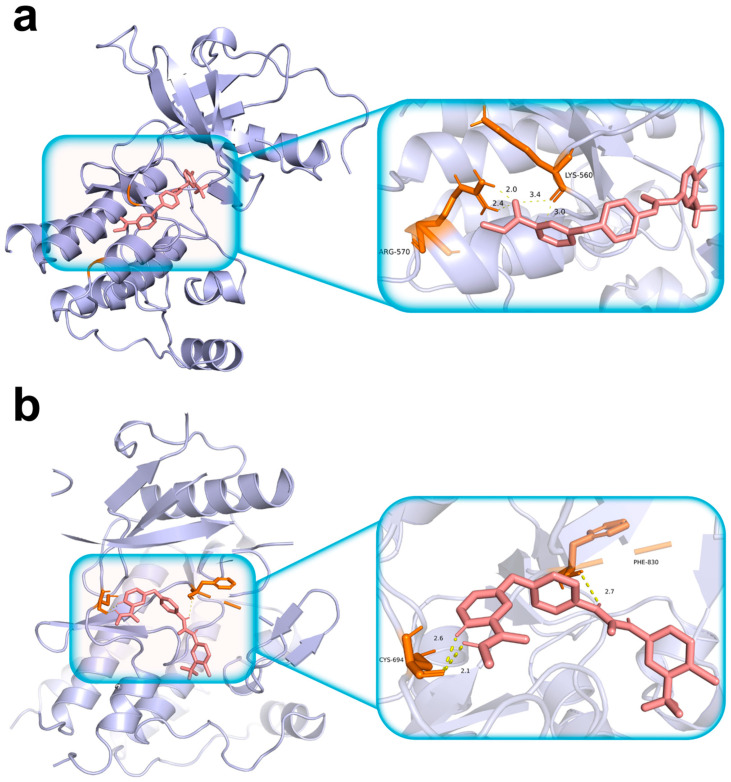
Structural validation of drug–target interactions by molecular docking. (**a**) Structural validation of drug–target interactions by molecular docking. (**a**) Sorafenib docking within the ATP-binding pocket of FGFR3. Left: overall ribbon representation of the FGFR3 kinase domain showing sorafenib occupying the ATP-binding site. Right: magnified view highlighting local residue interactions within the binding pocket. Hydrogen-bond interactions are observed with Lys560 and Arg570. (**b**) Sorafenib docking within the ATP-binding pocket of FLT3. Left: overall ribbon representation of the FLT3 kinase domain with sorafenib positioned in the ATP-binding site. Right: magnified view of the ligand–residue interaction interface, showing hydrogen-bond interactions with Cys694 and Phe830. Protein structures are shown as lavender ribbon cartoons; sorafenib is depicted as pink sticks; interacting amino-acid residues are shown as orange sticks; hydrogen bonds are represented by yellow dashed lines.

We used molecular docking to characterize sorafenib binding to the TIHS-prioritized targets *FGFR3* and *FLT3* (Table S7). Both complexes showed binding energies below −7 kcal/mol, and refined poses had docking scores of −8.646 kcal/mol for *FGFR3* and −8.383 kcal/mol for *FLT3* (Figure 6; Table S8). In *FGFR3* (Figure 6a), sorafenib engaged key residues within the ATP-binding domain, including Lys560 and Arg570, forming hydrogen bonds with bond lengths of 3.0 Å and 2.4 Å, respectively. In *FLT3* (Figure 6b), sorafenib established hydrogen bonds with Cys694 and Phe830, with bond distances of 2.1 Å and 2.7 Å, respectively. These docking results support the structural plausibility of sorafenib binding to both *FGFR3* and *FLT3*, consistent with TIHS-predicted target engagement across kinases.

#### 3.3.6. Molecular Dynamics Simulation

**Figure 7:**
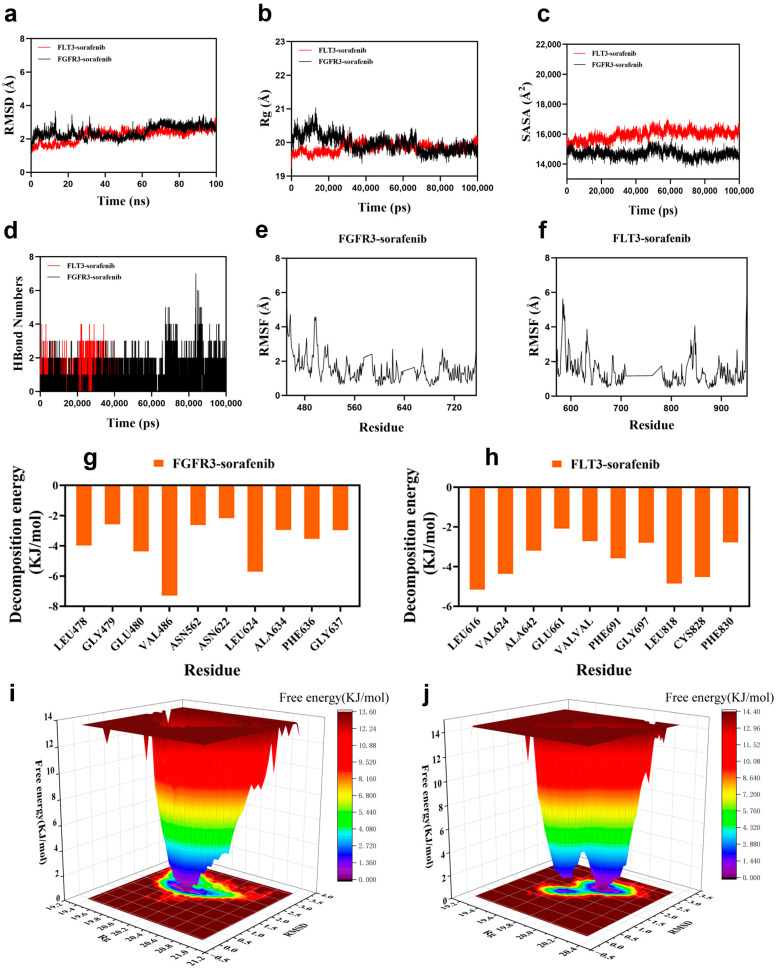
Molecular dynamics simulation of sorafenib binding to *FGFR3* and *FLT3*. (**a**) Root-mean-square deviation (RMSD) trajectories of *FGFR3*–sorafenib and *FLT3*–sorafenib complexes over 100 ns. (**b**) Radius of gyration (Rg) profiles indicating structural compactness of the complexes. (**c**) Solvent-accessible surface area (SASA) variations throughout the simulation period. (**d**) Time-dependent hydrogen bond counts between sorafenib and each target protein. (**e**,**f**) Root-mean-square fluctuation (RMSF) of amino acid residues in *FGFR3* (**e**) and *FLT3* (**f**), reflecting residue-level flexibility. (**g**,**h**) Per-residue binding energy contributions from molecular mechanics Poisson–Boltzmann surface area (MM/PBSA) calculations for *FGFR3* (**g**) and *FLT3* (**h**) complexes (**i**,**j**) Free energy landscape (FEL) plots for *FGFR3*–sorafenib (**i**) and *FLT3*–sorafenib (**j**) complexes using RMSD and Rg as reaction coordinates. Blue basins represent global energy minima, indicative of stable conformational states for each complex during the simulation.

We then examined the stability of these complexes using 100-nanosecond molecular dynamics simulations following molecular docking (Section 2.8). Key structural and energetic parameters were monitored throughout the simulation to evaluate the stability and specificity of ligand binding (Figure 7a–j). Root-mean-square deviation (RMSD) analysis (Figure 7a) revealed that both complexes rapidly reached equilibrium after approximately 30 ns, maintaining average RMSD values of ~2.9 Å for *FGFR3* and ~2.8 Å for *FLT3*, indicative of stable protein–ligand assemblies. The radius of gyration (Rg, Figure 7b) and solvent-accessible surface area (SASA, Figure 7c) showed only minor fluctuations over time, suggesting that both complexes retained global compactness and experienced no major conformational rearrangements. Hydrogen-bond profiling (Figure 7d) demonstrated persistent intermolecular interactions, with sorafenib forming 2–3 hydrogen bonds on average in each complex, supporting its stable occupation of the ATP-binding pocket. Backbone root-mean-square fluctuation (RMSF, Figure 7e,f) further confirmed that most residues—especially those proximal to the ligand—remained relatively rigid, with fluctuations below 4 Å.

Energetically, MM/PBSA binding free energy calculations indicated strong affinities for both complexes (−143.0 kJ/mol for *FGFR3* and −132.6 kJ/mol for *FLT3*). Per-residue decomposition energy analysis (Figure 7g,h) identified clusters of residues within the ATP-binding clefts of each kinase as major contributors to sorafenib binding. Free energy landscape (FEL) analysis mapped the conformational energy states of each complex during the simulation (Figure 7i,j), revealing distinct energy minima for both *FGFR3*–sorafenib and *FLT3*–sorafenib complexes as blue basins in the two-dimensional landscapes. These minima indicate favorable and stable conformational states under extensive conformational sampling. The absence of large-scale RMSD drift or conformational transitions during the production phase further supports sustained residence within these dominant basins. Together, these MD simulations and FEL analyses provide atomistic support for the structural stability and energetic favorability of sorafenib binding to both *FGFR3* and *FLT3*.

#### 3.3.7. Validation of mRNA Expression Changes in Cell Lines

**Figure 8:**
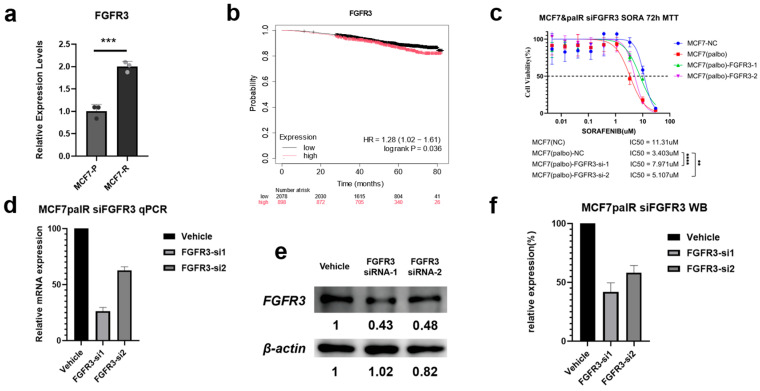
Functional validation of *FGFR3* as a TIHS-prioritized therapeutic hub in sorafenib response. (**a**) *FGFR3* mRNA expression in parental MCF7-P versus palbociclib-resistant MCF7-PR cells (RT-qPCR; two-tailed paired *t*-test). (**b**) Overall survival in the TCGA-BRCA cohort stratified by *FGFR3* expression (log-rank test). (**c**) Sorafenib dose–response curves in MCF7-PR cells transfected with non-targeting control or two independent *FGFR3*-targeting siRNAs; IC_50_ values were compared by extra sum-of-squares F-test. (**d**) RT-qPCR validation of *FGFR3* knockdown at the mRNA level in MCF7-PR cells for both siRNA constructs, relative to non-targeting control. (**e**) Western blot analysis of *FGFR3* protein levels after siRNA-mediated knockdown, with GAPDH as loading control. (**f**) Densitometric quantification of *FGFR3* protein normalized to GAPDH and expressed relative to non-targeting control. *p*-values: ** *p* < 0.01, *** *p* < 0.001, **** *p* < 0.0001.

We validated the mRNA expression changes in *FGFR3* and *FLT3* in our experimental models to confirm whether the gene expression alterations observed in sequencing were consistent with in vitro results. Quantitative PCR (qPCR) was performed on both parental MCF7 cells (MCF7-P) and palbociclib-resistant MCF7 cells (MCF7-PR). As shown in Figure 8a, *FGFR3* expression was consistently upregulated in MCF7-PR cells compared with parental MCF7 cells, with a log_2_ fold change of approximately 1.2, consistent with the sequencing data. This observation is consistent with *FGFR3* involvement in the resistant state. In contrast, *FLT3* exhibited much lower baseline expression levels in both MCF7-P and MCF7-PR cells. The Ct values for *FLT3* in MCF7-P (Ct ≈ 44) and MCF7-PR (Ct ≈ 38) were both relatively high, indicating low mRNA abundance. Although the fold change in *FLT3* expression appeared greater than that in the RNA-seq results, the absolute difference remained inconspicuous. Given these factors, *FLT3* was excluded from further functional analysis due to its low expression levels and inconsistent fold change in the resistant model.

#### 3.3.8. Prognostic Analysis of *FGFR3* Expression in TCGA-BRCA

Given the consistent upregulation of *FGFR3* in the resistant MCF7 cell lines, we proceeded to investigate the clinical relevance of *FGFR3* expression in breast cancer. We performed a survival analysis using the TCGA-BRCA dataset to determine whether high *FGFR3* expression correlates with poorer prognosis in breast cancer patients. As shown in Figure 8b, high *FGFR3* expression was significantly associated with poor survival outcomes (HR = 1.28, 95% CI = 1.02–1.61, log-rank *p* = 0.036). These findings suggest that *FGFR3* may not only serve as a molecular marker for resistance but also as a potential biomarker for poor prognosis in breast cancer. The association between high *FGFR3* expression and adverse clinical outcomes provides further justification for targeting *FGFR3* as a therapeutic strategy to reverse resistance and improve patient survival.

#### 3.3.9. Impact of *FGFR3* Knockdown on Sorafenib Sensitivity

Finally, we investigated the functional contribution of *FGFR3* to sorafenib response in the CDK4/6 inhibitor–resistant MCF7-PR model. Molecular and phenotypic readouts were used to link endogenous *FGFR3* activity to the TIHS-predicted sensitivity shift. Quantitative PCR confirmed that *FGFR3* mRNA expression was significantly upregulated in MCF7-PR cells compared with parental MCF7-P cells (*p* < 0.001; Figure 8a), consistent with the RNA-seq–derived fold change. In line with this, Kaplan–Meier analysis in the TCGA-BRCA cohort showed that higher *FGFR3* expression was associated with poorer overall survival (HR = 1.28, 95% CI: 1.02–1.61; log-rank *p* = 0.036; Figure 8b).

We then asked whether endogenous *FGFR3* activity contributes to the enhanced sorafenib sensitivity predicted by TIHS in resistant cells. MCF7-PR cells were transfected with two independent *FGFR3*-targeting siRNAs or a non-targeting control. RT-qPCR and Western blotting demonstrated efficient *FGFR3* depletion at both the mRNA and protein levels across both siRNAs relative to control (Figure 8d–f). Functionally, MTT assays performed 72 h after transfection revealed that the IC_50_ of sorafenib in unperturbed MCF7-PR cells was 3.403 μM, compared with 11.31 μM in MCF7-NC (negative control). Upon *FGFR3* knockdown, the sorafenib IC_50_ increased to 7.971 μM and 5.107 μM in the MCF7-PR-*FGFR3*-1 and MCF7-PR-*FGFR3*-2 groups, respectively (Figure 8c), indicating a partial loss of the resensitization effect following *FGFR3* silencing.

Together, these results show that *FGFR3* knockdown attenuates the growth-inhibitory effect of sorafenib in MCF7-PR cells, indicating that *FGFR3* is required for the full sorafenib response in this CDK4/6 inhibitor–resistant model. In line with its TIHS-defined hub status, these data provide experimental support that *FGFR3* and sorafenib form a functionally relevant drug–target pair in this resistance context.

## 4. Discussion

Following the experimental demonstration that *FGFR3* knockdown attenuates the growth-inhibitory effect of sorafenib in a CDK4/6 inhibitor–resistant MCF7 model, the present study places this drug–target interaction into a broader network context and evaluates its implications for resistance-state–aware drug repurposing. Given that sorafenib is a clinically approved multi-target kinase inhibitor with an established safety profile, this repurposing setting reduces translational uncertainty compared to de novo investigational compounds; however, efficacy and tolerability still warrant evaluation in the specific context of CDK4/6 inhibitor–resistant breast cancer. Together with multi-cohort computational analyses, these results indicate that a topology-integrated definition of hubness can converge on experimentally actionable drug–target pairs in a clinically relevant resistance setting.

To clarify the intended level of biological interpretation, we note that the present study was designed to validate the *predictive logic* of topology-defined vulnerability—namely, whether perturbation of a TIHS-prioritized hub alters drug response in a resistance-remodeled state—rather than to comprehensively delineate terminal downstream mechanisms of cell death or organelle stress. Sorafenib is a clinically established multi-kinase inhibitor that targets RAF-family kinases and multiple receptor tyrosine kinases, including VEGFRs and PDGFRβ, and such signaling breadth may constrain compensatory kinase-module rewiring that often accompanies resistance [43]. In ER^+^ breast-cancer models, aberrant *FGFR* signaling has been causally implicated in reduced sensitivity to endocrine therapy plus CDK4/6 inhibition, supporting the plausibility that *FGFR*-family nodes can become influential under CDK4/6i pressure [44]. Accordingly, our in vitro assays were focused on establishing hub-level functional dependency (*FGFR3*) in the observed sorafenib response, while detailed mapping of apoptosis execution, mitochondrial dysfunction, ER-stress readouts, or combination-interaction surfaces represents an important extension for future mechanistic and translational refinement.

The present study proposes a reframing of how “hubness” is quantified and exploited in resistance networks, particularly in the clinically urgent setting of CDK4/6 inhibitor resistance in HR^+^ breast cancer. Classical definitions of biological network hubs—based on degree, betweenness, or related centrality measures—capture only partial aspects of network influence and often yield inconsistent hub lists across datasets or algorithms [10,45,46,47]. Building on prior attempts to integrate multiple metrics into composite hub scores [48], TIHS consolidates complementary topological features into a single, interpretable hubness measure, providing a quantitatively grounded way to identify network “fragility points.” This approach aligns with theoretical and experimental evidence that hub nodes are disproportionately essential to network integrity and cellular viability [49,50,51,52].

Importantly, TIHS operates in a mechanism-agnostic manner during computational prioritization. Experimental validation, however, requires gene-level testing to confirm functional relevance. The selection of *FGFR3* for downstream analysis therefore reflects a topology-prioritized candidate rather than a predefined mechanistic assumption.

Such a framework is particularly well suited to resistance scenarios encountered in precision oncology, where therapy resistance is increasingly recognized as a patient- and state-specific phenomenon rather than a uniform, generalizable process. Tumors progressing on the same therapy frequently exhibit multiple, concurrent, or shifting resistance mechanisms [53,54,55,56], making it unrealistic to define a single dominant driver within the limited clinical decision window. Traditional precision oncology workflows are typically mechanism-centric, pairing a specific mutation or pathway alteration with a targeted agent [57,58,59]. In contrast, TIHS does not require a priori specification of a dominant driver gene or a predefined bypass pathway. Instead, it focuses on the emergent topology of the resistance-state interactome, prioritizing structurally vulnerable hubs without presupposing which mechanisms are operative, thereby enabling resistance-state–aware drug repurposing as a quantitative triage strategy when mechanistic certainty is limited.

TIHS also differs conceptually from existing repositioning and network-based strategies. Expression-reversal paradigms such as the Connectivity Map and LINCS match disease signatures to compounds that induce opposing transcriptional profiles [11,60,61], while master-regulator–driven platforms such as OncoTarget and OncoTreat infer regulatory programs to nominate actionable drugs [14]. Other approaches leverage structure-based or machine-learning models to expand the drug–target search space [62,63]. These methods have demonstrated value within their respective scopes but often rely on robust expression signatures, curated regulatory models, or complex predictive architectures that may be difficult to translate directly into time-sensitive resistance settings. Rather than competing with these paradigms, TIHS operates at a complementary layer by quantifying resistance-state hubness on PPI networks and intersecting this information with established drug–target relationships, thereby generating experimentally testable repurposing hypotheses.

At the same time, several limitations must be acknowledged. Conceptually, a high TIHS indicates that a node is a structurally vulnerable hub, but it does not explain why targeting that node overcomes resistance. TIHS is intentionally mechanism-agnostic and static: it does not model dynamic feedback, pathway cross-talk, or network rewiring, all of which are known to shape resistance evolution [64,65]. Accordingly, TIHS is designed to nominate candidate hubs for experimental interrogation rather than to infer causal signaling hierarchies. In settings where parallel pathways or redundant hubs compensate, inhibition of a single top-ranked node may be insufficient. Practical limitations also arise from data availability. The derivation and validation of TIHS relied primarily on retrospective bulk transcriptomic datasets from GEO, which remain susceptible to cohort-specific biases and limited sample sizes despite standardized preprocessing and cross-cohort benchmarking. Experimental validation was restricted to a single in-house palbociclib-resistant MCF7 model, and no prospective animal or patient-derived intervention studies were performed. Consequently, the *FGFR3*–sorafenib relationship described here should be interpreted as a model-specific proof-of-concept, rather than as a universally established resistance mechanism. Extension of TIHS-guided hypotheses to additional resistant models and prospective study designs will be required before clinical implementation can be considered.

Beyond these conceptual and experimental limitations, practical implementation of TIHS must be considered within real-world translational constraints. In post-resistance clinical settings, resistance is often recognized under time-sensitive conditions, while the underlying biology may be heterogeneous, concurrent, or incompletely defined. Acquisition of resistance-state molecular profiles can itself be challenging, as paired sampling at progression is not always feasible and publicly available datasets remain limited and cohort-specific. These realities motivated the design of TIHS as a resistance-state–aware triage layer rather than a mechanism-resolving model. By operating on standard bulk transcriptomic inputs and publicly curated interaction and drug–target databases, the framework remains compatible with conventional academic computing environments. In practical deployment scenarios, data acquisition and standardized preprocessing are likely to represent greater bottlenecks than computational runtime. Accordingly, prospective clinical integration would require harmonized preprocessing pipelines and streamlined reporting workflows to ensure reproducible and timely prioritization outputs within clinically relevant decision windows.

To actively mitigate the uncertainties and noise inherent to these computational and data-driven limitations, the real-world application of TIHS necessitates the integration of strict false-positive control filters. While our study utilized a rigorous multi-cohort intersection to initially identify six candidates, transitioning this framework to single-patient datasets requires objective, standardized thresholds. By enforcing a target multiplicity threshold (≥2 targets), we effectively deprioritized single-target agents that are prone to bypass resistance. Furthermore, the integration of absolute expression levels served as a critical biological safeguard: although *FLT3* scored computationally as a topological hub, its negligible transcript abundance correctly flagged it as a biological false positive, efficiently directing our functional validation toward the highly expressed *FGFR3*. Similarly, applying pharmacokinetic boundaries successfully filtered out agents like quizartinib, which, despite exhibiting in vitro shifts, required concentrations far exceeding their clinical *C_max_*. This multi-tiered strategy—combining topological scoring, target count, expression abundance, and clinical feasibility—ensures a robust and reproducible screening pipeline even in single-dataset scenarios.

Ultimately, these limitations, along with our corresponding methodological safeguards, define the intended scope of TIHS rather than undermine its utility. Within this defined scope, the framework has notable translational implications. More broadly, this work addresses a common bottleneck in precision oncology, where high-dimensional molecular data must be translated into actionable hypotheses under conditions of heterogeneity, uncertainty, and limited time. By ranking existing drugs according to their coverage of resistance-state hubs, TIHS provides a quantitative triage layer that can, in principle, generate prioritized therapeutic hypotheses once resistance-state molecular data become available. Such outputs are intended to support multidisciplinary discussion and experimental or clinical prioritization, rather than to replace clinical judgment or guideline-based decision-making. By operating entirely within the space of established targets and approved or investigational agents, TIHS reframes post-resistance management as a constrained reoptimization problem rather than a de novo drug discovery task. Because the framework does not rely on tumor-type-specific mechanism models, it may be extensible to other resistance contexts, provided that high-quality resistance-state data and appropriate interaction maps are available. This network-centric perspective is consistent with emerging evidence that targeting structural vulnerabilities may offer a complementary strategy to mutation-focused interventions [66,67,68].

## 5. Conclusions

In summary, this study establishes the TIHS as a mechanism-agnostic, resistance-state–aware framework that bridges transcriptomic network remodeling with experimentally testable drug–target hypotheses. By formalizing hubness as a quantitative, topology-integrated property and intersecting it with curated drug–target interaction knowledge, TIHS enables principled prioritization of clinically available agents without reliance on predefined resistance mechanisms. Applied to CDK4/6 inhibitor–resistant HR^+^ breast cancer, the framework consistently converged on sorafenib as a high-priority candidate and supported *FGFR3* as a functionally relevant network hub through in vitro validation, demonstrating that targeting topology-defined hubs can yield actionable repurposing strategies in resistant disease states.

Beyond this specific application, TIHS is intended as a quantitative decision-support and prioritization tool rather than a replacement for clinical judgment. In precision oncology settings characterized by heterogeneous and incompletely defined resistance mechanisms, the framework narrows the therapeutic search space while simultaneously generating hypotheses for downstream mechanistic investigation. Although this study focuses on CDK4/6 inhibitor resistance in HR^+^ breast cancer, the underlying principle of topology-defined vulnerability may be conditionally extensible to other resistance contexts, provided that high-quality resistance-state molecular data and appropriate interaction maps are available.

## Figures and Tables

**Table 1:** Validation results of each dataset and ranking information of drugs to be tested.

Dataset	Cell Lines	Drugs	Actual Effect	Predictive Accuracy	Relative Ranking	Relative Ranking (%)
GSE62504	HCC827-BR1	Afatinib	Resistance	Yes	7/130	5.38%
GSE62504	HCC827-BR1	Dasatinib	Sensitive	Yes	2/130	1.54%
GSE62504	HCC827-BR2	Afatinib	Resistance	Yes	6/118	5.08%
GSE62504	HCC827-BR2	Dasatinib	Sensitive	No	NA	NA
GSE129221	PC9	Gefitinib	Resistance	Yes	30/149	20.13%
GSE129221	PC9	Apatinib	Sensitive	No	Not observed	NA
GSE200029	T47D	Tamoxifen	Resistance	Yes	6/172	3.49%
GSE200029	T47D	Erdatinib	Sensitive	Yes	13/172	7.56%
GSE268699	MCF7	Palbociclib	Resistance	Yes	52/84	61.90%
GSE268699	MCF7	Fulvestrant	Resistance	Yes	3/188	1.60%
GSE268699	T47D	Palbociclib	Resistance	No	NA	NA
GSE268699	T47D	Fulvestrant	Resistance	No	Not observed	NA

## Data Availability

All public datasets analyzed in this study are available from GEO under the accessions GSE62504, GSE129221, GSE200029, GSE268699, GSE222367 and GSE229235. Source data underlying the plots and summary statistics (DEG tables, ranked drug lists, topology weights and validation metrics) are provided in Supplementary Data. The laboratory-generated RNA-seq data from the MCF7-PR model will be deposited in GEO and made public upon acceptance; during peer review, anonymized access can be provided by the corresponding authors on reasonable request and with institutional approval. Raw data are available from the corresponding author upon reasonable request. All custom analysis code necessary to reproduce the manuscript’s results is publicly available on GitHub at https://github.com/ZombieScript/drug_sensitivity_scoring, accessed on 27 February 2026. This repository includes fully annotated R scripts for differential expression analysis (DESeq2 or limma), PPI network topology scoring, drug–target integration, and statistical validation, along with a comprehensive README and an explicit license file (MIT).

## Supplementary Materials

The following supporting information can be downloaded at: https://www.mdpi.com/article/10.3390/biomedicines14030592/s1, Table S1: Summary of the accuracy of 5 types of topological scoring and equal-weighted comprehensive scoring. Table S2: McNemar’s test results comparing equal-weighted composite scores with single topological metrics. Table S3: Actual weights of the five topological attributes derived from PCA-shift analysis in each test dataset. Table S4: Chi-square tests of equal-weight assumption and cross-dataset homogeneity for topology metric weights. Table S5: Consistency of weight distributions across datasets relative to the overall mean. Table S6: List of predictive drugs that are effective in the CDK4/6 inhibitor dataset after drug target mapping. Table S7: Details of Sorafenib Matching Results. Table S8: Summary of the binding energy and hydrogen bond details regarding the stable binding mode of Sorafenib with the two targets. Table S9: Primer and siRNA sequences. Figure S1: Volcano plots of differentially expressed genes (DEGs) between drug-sensitive and resistant models across datasets. (a, b) DEGs in MCF7 and T47D parental vs. palbociclib-resistant cells (GSE222367). (c) DEGs in the PDX model (GSE229235). (d) DEGs in the external MCF7-resistant model derived in-house. Dashed horizontal lines indicate the significance threshold (adjusted *p* < 0.05); vertical lines represent fold-change cutoffs (|log_2_FC| ≥ 1). Genes up- and down-regulated are highlighted accordingly. Figure S2: Cross-model transcriptomic heterogeneity and stability of resistance-associated shift profiles. PCA, bootstrap stability, and correlation-based clustering of resistance-associated Log_2_FC shift profiles. (a) Principal component analysis of gene-level Log_2_FC resistance shift profiles across four CDK4/6i-resistant models using intersected genes. (b) Bootstrap PCA distributions (n = 500 gene-resampling iterations) demonstrating stability of model separation in principal component space. (c) Pairwise Pearson correlation heatmap of resistance shift vectors, illustrating molecular divergence among CDK4/6i resistance models. Figure S3: MCF7 drug validation results.

## Abbreviations

The following abbreviations are used in this manuscript:

TIHS	Topology-Integrated Hubness Score
CDK4/6i	Cyclin-Dependent Kinase 4/6 Inhibitors
HR^+^	Hormone Receptor–Positive
PPI	Protein–Protein Interaction
DEG	Differentially Expressed Gene
GEO	Gene Expression Omnibus
PDX	Patient-Derived Xenograft
TCGA-BRCA	The Cancer Genome Atlas Breast Cancer Cohort
RNA-seq	RNA Sequencing
siRNA	Small Interfering RNA
IC_50_	Half-Maximal Inhibitory Concentration
EC_50_	Half-Maximal Effective Concentration
MCC	Maximal Clique Centrality
EPC	Edge Percolated Component
DSS	Drug Sensitivity Score
RMSD	Root Mean Square Deviation
RMSF	Root Mean Square Fluctuation
Rg	Radius of Gyration
SASA	Solvent-Accessible Surface Area
MM/PBSA	Molecular Mechanics/Poisson–Boltzmann Surface Area
FEL	Free Energy Landscape
Cmax	maximum plasma concentration
*FGFR*	Fibroblast Growth Factor Receptor
HR	Hazard ratio
CI	Confidence interval
KMplot	Kaplan–Meier Plotter
