# Supplementary material for: A Tumor-Agnostic, Topology-Informed Scoring Framework for Drug Repurposing: Application to CDK4/6 Inhibitor Resistance in HR+ Breast Cancer
Source: Biomedicines. 2026 Mar 6;14(3):592. doi: 10.3390/biomedicines14030592 (PMC13024279; doi:10.3390/biomedicines14030592)
Supplement: Supplementary file 1 [file biomedicines-14-00592-s001.zip › biomedicines-4141857-supplementary.pdf]

Supplementary Data

Table S1 Summary of the accuracy of 5 types of topological scoring and equal-weighted comprehensive scoring

Topo_Metrics	Dataset	Cell Lines	Drugs	Actual Effect	Predictive Accuracy	Relative Ranking	Relative Ranking(%)
Degree	GSE62504	HCC827-BR1	Afatinib	Resistance	yes	7/130	5.38%
Degree	GSE62504	HCC827-BR1	Dasatinib	Sensitive	no	99/130	76.15%
Degree	GSE62504	HCC827-BR2	Afatinib	Resistance	yes	6/118	5.08%
Degree	GSE62504	HCC827-BR2	Dasatinib	Sensitive	no	92/118	77.97%
Degree	GSE129221	PC9	Gefitinib	Resistance	no	113/149	75.84%
Degree	GSE129221	PC9	Apatinib	Sensitive	Not Observed	Not Observed	NA
Degree	GSE200029	T47D	Tamoxifen	Resistance	no	167/172	97.09%
Degree	GSE200029	T47D	Erdafitinib	Sensitive	yes	26/172	15.12%
Degree	GSE268699	MCF7	Palbociclib	Resistance	yes	52/84	61.90%
Degree	GSE268699	MCF7	Fulvestrant	Resistance	yes	2/188	1.06%
Degree	GSE268699	T47D	Palbociclib	Resistance	no	66/99	66.67%
Degree	GSE268699	T47D	Fulvestrant	Resistance	Not Observed	Not Observed	NA
Betweenness	GSE62504	HCC827-BR1	Afatinib	Resistance	yes	6/130	4.62%
Betweenness	GSE62504	HCC827-BR1	Dasatinib	Sensitive	yes	2/130	1.54%
Betweenness	GSE62504	HCC827-BR2	Afatinib	Resistance	yes	8/118	6.78%
Betweenness	GSE62504	HCC827-BR2	Dasatinib	Sensitive	yes	2/118	1.69%
Betweenness	GSE129221	PC9	Gefitinib	Resistance	yes	113/149	75.84%
Betweenness	GSE129221	PC9	Apatinib	Sensitive	Not Observed	Not Observed	NA
Betweenness	GSE200029	T47D	Tamoxifen	Resistance	no	168/172	97.67%
Betweenness	GSE200029	T47D	Erdafitinib	Sensitive	yes	20/172	11.63%
Betweenness	GSE268699	MCF7	Palbociclib	Resistance	yes	52/84	61.90%

Betweenness	GSE268699	MCF7	Fulvestrant	Resistance	yes	3/188	1.60%
Betweenness	GSE268699	T47D	Palbociclib	Resistance	no	56/99	56.57%
Betweenness	GSE268699	T47D	Fulvestrant	Resistance	Not Observed	Not Observed	NA
MCC	GSE62504	HCC827-BR1	Afatinib	Resistance	yes	8/130	6.15%
MCC	GSE62504	HCC827-BR1	Dasatinib	Sensitive	yes	6/130	4.62%
MCC	GSE62504	HCC827-BR2	Afatinib	Resistance	yes	5/118	4.24%
MCC	GSE62504	HCC827-BR2	Dasatinib	Sensitive	yes	5/118	4.24%
MCC	GSE129221	PC9	Gefitinib	Resistance	no		
MCC	GSE129221	PC9	Apatinib	Sensitive	Not Observed	Not Observed	NA
MCC	GSE200029	T47D	Tamoxifen	Resistance	no		
MCC	GSE200029	T47D	Erdafitinib	Sensitive	yes	44/172	25.58%
MCC	GSE268699	MCF7	Palbociclib	Resistance	yes	30/84	35.71%
MCC	GSE268699	MCF7	Fulvestrant	Resistance	yes	8/188	1.06%
MCC	GSE268699	T47D	Palbociclib	Resistance	no		
MCC	GSE268699	T47D	Fulvestrant	Resistance	Not Observed	Not Observed	NA
EPC	GSE62504	HCC827-BR1	Afatinib	Resistance	yes	6/130	4.62%
EPC	GSE62504	HCC827-BR1	Dasatinib	Sensitive	no	128/130	98.46%
EPC	GSE62504	HCC827-BR2	Afatinib	Resistance	yes	6/118	5.08%
EPC	GSE62504	HCC827-BR2	Dasatinib	Sensitive	no	116/118	98.31%
EPC	GSE129221	PC9	Gefitinib	Resistance	no		
EPC	GSE129221	PC9	Apatinib	Sensitive	Not Observed	Not Observed	NA
EPC	GSE200029	T47D	Tamoxifen	Resistance	no		
EPC	GSE200029	T47D	Erdafitinib	Sensitive	yes	44/172	25.58%
EPC	GSE268699	MCF7	Palbociclib	Resistance	yes	37/84	44.05%
EPC	GSE268699	MCF7	Fulvestrant	Resistance	yes	2/188	1.06%
EPC	GSE268699	T47D	Palbociclib	Resistance	no		

EPC	GSE268699	T47D	Fulvestrant	Resistance	Not Observed	Not Observed	NA
Eigenvector	GSE62504	HCC827-BR1	Afatinib	Resistance	yes	9/130	6.92%
Eigenvector	GSE62504	HCC827-BR1	Dasatinib	Sensitive	no	93/130	71.54%
Eigenvector	GSE62504	HCC827-BR2	Afatinib	Resistance	yes	6/118	5.08%
Eigenvector	GSE62504	HCC827-BR2	Dasatinib	Sensitive	no	98/118	83.05%
Eigenvector	GSE129221	PC9	Gefitinib	Resistance	no		
Eigenvector	GSE129221	PC9	Apatinib	Sensitive	Not Observed	Not Observed	NA
Eigenvector	GSE200029	T47D	Tamoxifen	Resistance	no		
Eigenvector	GSE200029	T47D	Erdafitinib	Sensitive	yes	20/172	11.63%
Eigenvector	GSE268699	MCF7	Palbociclib	Resistance	yes	49/84	61.90%
Eigenvector	GSE268699	MCF7	Fulvestrant	Resistance	yes	1/188	0.53%
Eigenvector	GSE268699	T47D	Palbociclib	Resistance	no		
Eigenvector	GSE268699	T47D	Fulvestrant	Resistance	Not Observed	Not Observed	NA
Equal-weighted sum of five metrics	GSE62504	HCC827-BR1	Afatinib	Resistance	yes	7/130	5.38%
Equal-weighted sum of five metrics	GSE62504	HCC827-BR1	Dasatinib	Sensitive	yes	2/130	1.54%
Equal-weighted sum of five metrics	GSE62504	HCC827-BR2	Afatinib	Resistance	yes	6/118	5.08%
Equal-weighted sum of five metrics	GSE62504	HCC827-BR2	Dasatinib	Sensitive	yes	11/118	9.32%
Equal-weighted sum of five metrics	GSE129221	PC9	Gefitinib	Resistance	yes	30/149	20.13%
Equal-weighted sum of five metrics	GSE129221	PC9	Apatinib	Sensitive	Not Observed	Not Observed	NA
Equal-weighted sum of five metrics	GSE200029	T47D	Tamoxifen	Resistance	yes	5/172	2.91%
Equal-weighted sum of five metrics	GSE200029	T47D	Erdafitinib	Sensitive	yes	13/172	7.56%
Equal-weighted sum of five metrics	GSE268699	MCF7	Palbociclib	Resistance	yes	54/84	64.29%
Equal-weighted sum of five metrics	GSE268699	MCF7	Fulvestrant	Resistance	yes	3/188	1.60%
Equal-weighted sum of five metrics	GSE268699	T47D	Palbociclib	Resistance	no	62/99	62.63%
Equal-weighted sum of five metrics	GSE268699	T47D	Fulvestrant	Resistance	Not Observed	Not Observed	NA

Table S2 McNemar’s test results comparing equal-weighted composite scores with single topological metrics

Prediction accuracy (equal-weighted vs single-metric)					
**metric**	**n11**	**n10**	**n01**	**n00**	**p_mcnemar_one_sided**
Degree	5	4	0	3	0.0625
EPC	5	4	0	3	0.0625
Eigenvector	5	4	0	3	0.0625
MCC	7	2	0	3	0.25
Betweenness	8	1	0	3	0.5
Overall	30	15	0	15	3.05E-05
**Top10 enrichment accuracy (equal-weighted vs single-metric)**					
**metric**	**n11**	**n10**	**n01**	**n00**	**p_mcnemar_one_sided**
Degree	2	5	0	5	0.03125
EPC	2	5	0	5	0.03125
Eigenvector	2	5	0	5	0.03125
MCC	4	3	0	5	0.125
Betweenness	5	2	0	5	0.25
Overall	15	20	0	25	9.54E-07
**Top30 enrichment accuracy (equal-weighted vs single-metric)**					
**metric**	**n11**	**n10**	**n01**	**n00**	**p_mcnemar_one_sided**
Degree	3	5	0	4	0.03125
EPC	3	5	0	4	0.03125
Eigenvector	3	5	0	4	0.03125
MCC	5	3	0	4	0.125
Betweenness	6	2	0	4	0.25
Overall	20	20	0	20	9.54E-07

The table summarizes pairwise McNemar’s tests between the equal-weighted composite score and each single topological metric (Degree, Betweenness, Eigenvector, MCC, EPC) across different evaluation criteria. The counts (n11, n10, n01, n00) indicate the number of datasets where both approaches were correct (n11), equal-weighted was correct while single-metric was not (n10), equal-weighted was incorrect while single-metric was correct (n01), or both

were incorrect (n00). One-sided McNemar’ s test p-values are reported under the alternative hypothesis that the equal-weighted method performs better.
Results are shown for:
(a) Prediction accuracy,
(b) Top10 ranking enrichment, and
(c) Top30 ranking enrichment.
In the overall analysis, the equal-weighted strategy significantly outperformed single metrics (accuracy $p=3.0\times10^{-5}$; Top10 $p=9.5\times10^{-7}$; Top30 $p=9.5\times10^{-7}$).

Table S3 Actual weights of the five topological attributes derived from PCA-shift analysis in each test dataset.

Datasets	cell_line	Degree_shifted	Betweenness_shifted	Eigenvector_shifted	MCC_shifted	EPC_shifted
GSE62504	BR1	0.63515	0.32381	0.03062	0.01026	0.00016
GSE62504	BR2	0.72493	0.20647	0.05809	0.01040	0.00011
GSE129221	PC9	0.73782	0.17419	0.07598	0.01193	0.00008
GSE200029	T47D	0.73497	0.18762	0.06707	0.01029	0.00005
GSE268699	MCF7-CDK4&6	0.60195	0.36780	0.01994	0.01009	0.00022
GSE268699	MCF7-fulvestrant	0.59712	0.35998	0.03722	0.00565	0.00003
GSE268699	T47D-CDK4&6	0.58897	0.37655	0.02719	0.00714	0.00015
GSE268699	T47D-fulvestrant	0.58773	0.37103	0.03300	0.00809	0.00015

The table reports the relative weights assigned to each topological attribute (Degree, Betweenness, Eigenvector, MCC, EPC) in different datasets and cell line contexts. These weights were obtained after Z-score normalization and positive shifting, followed by PCA-based variance contribution analysis. The results demonstrate that Degree and Betweenness consistently dominate the composite importance score, whereas Eigenvector, MCC, and EPC contribute minimally.

Table S4 Chi-square tests of equal-weight assumption and cross-dataset homogeneity for topology metric weights

ID	Degree	Betweenness	Eigenvector	MCC	EPC	ChiSq	df	p.value
GSE62504 | BR1	0.63515	0.32381	0.03062	0.01026	0.00016	154655.6581	4	0
GSE62504 | BR2	0.72493	0.20647	0.05809	0.01040	0.00011	185817.9930	4	0
GSE129221 | PC9	0.73782	0.17419	0.07598	0.01193	0.00008	190317.9001	4	0
GSE200029 | T47D	0.73497	0.18762	0.06707	0.01029	0.00005	189993.2184	4	0
GSE268699 | MCF7-CDK4&6	0.60195	0.36780	0.01994	0.01009	0.00022	149060.0513	4	0
GSE268699 | MCF7-fulvestrant	0.59712	0.35998	0.03722	0.00565	0.00003	143777.5733	4	0
GSE268699 | T47D-CDK4&6	0.58897	0.37655	0.02719	0.00714	0.00015	144732.9308	4	0
GSE268699 | T47D-fulvestrant	0.58773	0.37103	0.03300	0.00809	0.00015	142122.1422	4	0
Cross-set weight test results	0.65108	0.29593	0.04363	0.00924	0.00012	31445	28	< 2.2e-16

Table S4 shows the χ^2 goodness-of-fit tests for each dataset (null hypothesis: equal contribution of five metrics, 20% each) and the χ^2 homogeneity test across all datasets (null hypothesis: identical weight distributions across datasets). In all cases, the equal-weight assumption was rejected ($p < 0.0001$). Moreover, the across-dataset test also indicated significant heterogeneity (χ^2=31445, df=28, p<2.2e-16). These results support the necessity of using weighted rather than equal integration in subsequent analyses.

Table S5 Consistency of weight distributions across datasets relative to the overall mean

ID	Cosine_to_Mean	Pearson_to_Mean
GSE62504 | BR1	1.00	1.00
GSE62504 | BR2	0.99	0.98
GSE129221 | PC9	0.98	0.97
GSE200029 | T47D	0.98	0.98
GSE268699 | MCF7-CDK4&6	0.99	0.99
GSE268699 | MCF7-fulvestrant	0.99	0.99
GSE268699 | T47D-CDK4&6	0.99	0.98
GSE268699 | T47D-fulvestrant	0.99	0.98

For each dataset, cosine similarity and Pearson correlation were calculated between its weight vector (Degree, Betweenness, Eigenvector, MCC, EPC) and the across-dataset mean weight vector. All datasets showed high similarity (cosine ≥ 0.98, Pearson r ≥ 0.97), indicating that the overall ranking of metric contributions (Degree > Betweenness ≫ Eigenvector, MCC, EPC) is stable across datasets despite minor proportional differences.

Table S6 List of predictive drugs that are effective in the CDK4/6 inhibitor dataset after drug target mapping

MCF7_GSE222367	T47D_GSE222367	PDX_GSE229235	MCF7_Lab
DASATINIB	DASATINIB	DASATINIB	TOZASERTIB
CEDIRANIB	ISOLIQUIRITIGENIN	TAMOXIFEN	NILOTINIB
TOZASERTIB	SUNITINIB	VANDETANIB	CERITINIB
VANDETANIB	SORAFENIB	ENTRECTINIB	PONATINIB
DUVELISIB	DIETHYLSTILBESTROL	CEDIRANIB	GEFITINIB
NILOTINIB	IMATINIB	ERLOTINIB	ERLOTINIB
CERITINIB	MITOXANTRONE	PONATINIB	SORAFENIB
BOSUTINIB	PAZOPANIB	PAZOPANIB	CABOZANTINIB
IBRUTINIB	NILOTINIB	SORAFENIB	OSIMERTINIB
TIRBANIBULIN	CELECOXIB	PALBOCICLIB	AFATINIB
PAZOPANIB	DAUNORUBICIN	BOSUTINIB	CRIZOTINIB
SUNITINIB	DOXORUBICIN	TOZASERTIB	LAPATINIB
SORAFENIB	SELINEXOR	NILOTINIB	NERATINIB
DIETHYLSTILBESTROL	ETHINYL ESTRADIOL	IDELALISIB	MIDOSTAURIN
ISOLIQUIRITIGENIN	PACLITAXEL	ABEMACICLIB	VENETOCLAX
MITOXANTRONE	MASOPROCOL	GEFITINIB	QUIZARTINIB
PACLITAXEL	QUIZARTINIB	ISOLIQUIRITIGENIN	DIETHYLSTILBESTROL
IMATINIB	BOSUTINIB	CABOZANTINIB	PAZOPANIB
ALECTINIB	PONATINIB	QUIZARTINIB	ERDAFITINIB
ETHINYL ESTRADIOL	EPIRUBICIN	DINACICLIB	TOFACITINIB
BELINOSTAT	BELINOSTAT	ALVOCIDIB	NINTEDANIB
DOXORUBICIN	BEXAROTENE	OSIMERTINIB	LORLATINIB
QUIZARTINIB	AMSACRINE	PACLITAXEL	MEDROXYPROGESTERONE ACETATE
PHENTOLAMINE	SIROLIMUS	FULVESTRANT	INFIGRATINIB
CRIZOTINIB	IDELALISIB	TOFACITINIB	FUTIBATINIB
INDIRUBIN	VINBLASTINE	AFATINIB	AMSACRINE

VINBLASTINE	VINCRISTINE	RALOXIFENE HYDROCHLORIDE	DAUNORUBICIN
VINBLASTINE SULFATE	CERITINIB	RIBOCICLIB	MASOPROCOL
DOCETAXEL	REGORAFENIB	RUXOLITINIB	RUXOLITINIB
VINCRISTINE	PEXIDARTINIB	FILGOTINIB	UPADACITINIB
VINORELBINE	TRETINOIN	ALPELISIB	BARICITINIB
EMBELIN	VINBLASTINE SULFATE	MITOXANTRONE	FILGOTINIB
DINACICLIB	DOCETAXEL	DOXORUBICIN	PLERIXAFOR
LORLATINIB	VINORELBINE	DUVELISIB	ENZALUTAMIDE
	TIRBANIBULIN	LAPATINIB	SIROLIMUS
	TRANYLCYPROMINE HYDROCHLORIDE	CISPLATIN	LEFLUNOMIDE
	CROCIN	CERITINIB	THIOTEPA
	IDARUBICIN	BORTEZOMIB	DINACICLIB
	ALITRETINOIN	BELINOSTAT	PHENTOLAMINE
	THIOGUANINE	BARICITINIB	PEXIDARTINIB
	AMINOGLUTETHIMIDE	ALECTINIB	FLUTAMIDE
	EVEROLIMUS	IBRUTINIB	CHLOROPYRAMINE
	SORAFENIB TOSYLATE	ACALABRUTINIB	IDARUBICIN
	AMRUBICIN	NERATINIB	BEXAROTENE
	MELPHALAN	INDIRUBIN	GILTERITINIB
	ENASIDENIB	ADAGRASIB	ALVOCIDIB
	PHENTOLAMINE	ASCIMINIB	APALUTAMIDE
	CAMPTOTHECIN	ROCILETINIB	CAMPTOTHECIN
		DACOMITINIB	VEMURAFENIB
		AFATINIB DIMALEATE	
		CRIZOTINIB	
		ULIPRISTAL ACETATE	
		SELINEXOR	

IXAZOMIB
VENETOCLAX
AXITINIB
DABRAFENIB
COPANLISIB
TRILACICLIB
LORLATINIB
INFIGRATINIB
UPADACITINIB
BICALUTAMIDE
CARFILZOMIB
MASOPROCOL
ENZALUTAMIDE
NILUTAMIDE
ZANUBRUTINIB
CELECOXIB
APALUTAMIDE
ENASIDENIB
VINBLASTINE
VINCRISTINE
ENTINOSTAT
GILTERITINIB
METHOTREXATE
VORINOSTAT
PLERIXAFOR
VINBLASTINE SULFATE
DOCETAXEL
VINORELBINE
TIRBANIBULIN

SIROLIMUS
PHENTOLAMINE
PANOBINOSTAT
EMBELIN
FLUPIRTINE MALEATE
THIOGUANINE
MELPHALAN
MYCOPHENOLIC ACID
AMSACRINE
THIOTEPA
PEMETREXED

Table S7 Details of Sorafenib Matching Results

Gene	Target Importance	Standard Value(nM)	Log_2FC	Effective Type
BLK	2.37	794.33	-6.15	Suppress
EPHA2	7.32	794.33	-3.56	Suppress
LYN	13.20	398.11	-4.80	Suppress
**FGFR3**	7.50	1258.93	**1.20**	Suppress
**FLT3**	5.41	2	**2.75**	Suppress
MAOA	4.63	8258.3	-5.21	Suppress
KIT	19.18	11	-1.62	Suppress

Table S8 Summary of the binding energy and hydrogen bond details regarding the stable binding mode of Sorafenib with the two targets

Mode Number	Target Name	Binding Energy (kcal/mol)	Amino Acid Residue	H-bonding Distance
**1**	FGFR3	-8.646	Arginine-570	2 Å<br>4.3 Å
			Lysine-560	3 Å<br>3.4 Å
**12**	FLT3	-8.383	Cysteine-694	2.1 Å<br>2.6 Å
			Phenylalanine-830	2.7 Å

Table S9 Primer and siRNA sequences.

Primers	Sequences
GAPDH-homo(Sequences-F)	GGAGCGAGATCCCTCCAAAAT
GAPDH-homo(Sequences-R)	GGCTGTTGTCATACTTCTCATGG
FGFR3-homo(Sequences-F)	AGGATGCCTGCATACACACTGC
FGFR3-homo(Sequences-R)	ACACCCTACGTTACCGTGCTCAAG
FLT3-homo(Sequences-F)	AGTCAGAAGGGACTGGCTCC
FLT3-homo(Sequences-R)	GAGTGCTGCTTAGCAGATTACC
**Sequences for siRNAs**	
si-FGFR3#1(Sequences-F)	CCACCGACAAGGAGCUAGATT
si-FGFR3#1(Sequences-R)	UCUAGCUCCUUGUCGGUGGTT
si-FGFR3#2(Sequences-F)	CGCGUACUGUGCCACUUCATT
si-FGFR3#2(Sequences-R)	UGAAGUGGCACAGUACGCGTT

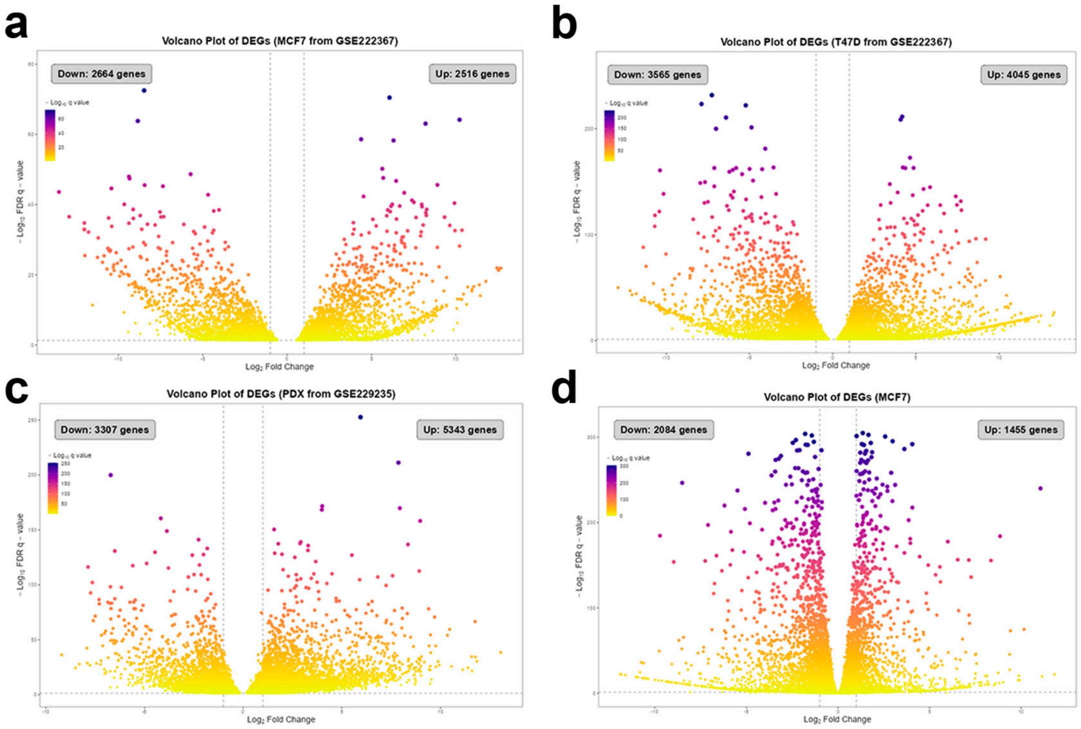

Figure S1. Volcano plots of differentially expressed genes (DEGs) between drug-sensitive and resistant models across datasets. (a, b) DEGs in MCF7 and T47D parental vs. palbociclib-resistant cells (GSE222367). (c) DEGs in the PDX model (GSE229235). (d) DEGs in the external MCF7-resistant model derived in-house. Dashed horizontal lines indicate the significance threshold (adjusted $p < 0.05$); vertical lines represent fold-change cutoffs ($|log_2FC| \geqslant 1$). Genes up- and down-regulated are highlighted accordingly.

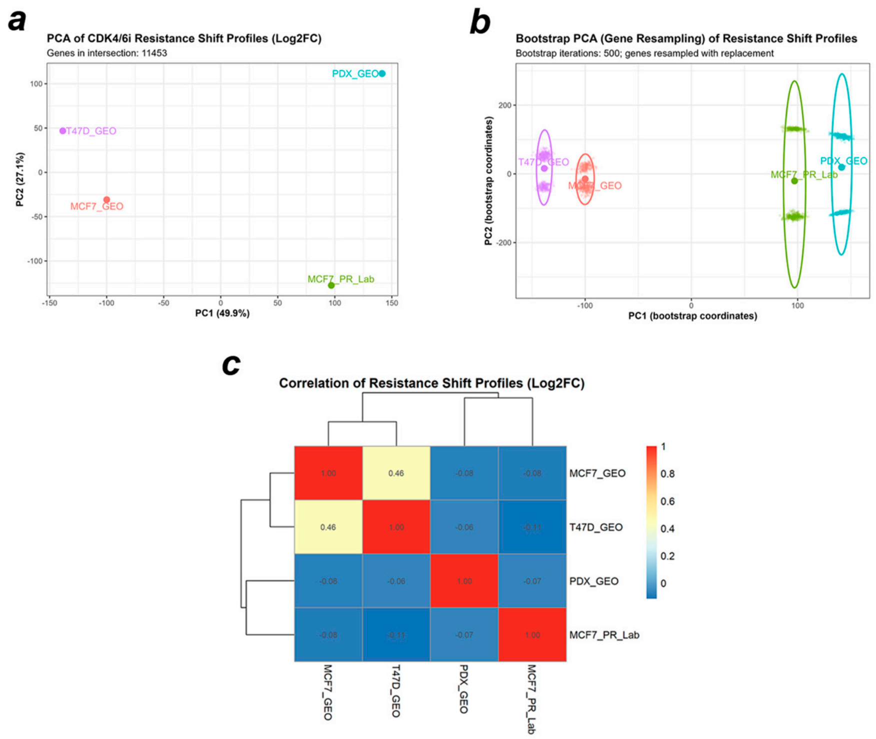

Figure S2. Cross-model transcriptomic heterogeneity and stability of resistance-associated shift profiles. PCA, bootstrap stability, and correlation-based clustering of resistance-associated Log_2FC shift profiles. (a) Principal component analysis of gene-level Log_2FC resistance shift profiles across four CDK4/6i-resistant models using intersected genes. (b) Bootstrap PCA distributions (n = 500 gene-resampling iterations) demonstrating stability of model separation in principal component space. (c) Pairwise Pearson correlation heatmap of resistance shift vectors, illustrating molecular divergence among CDK4/6i resistance models.

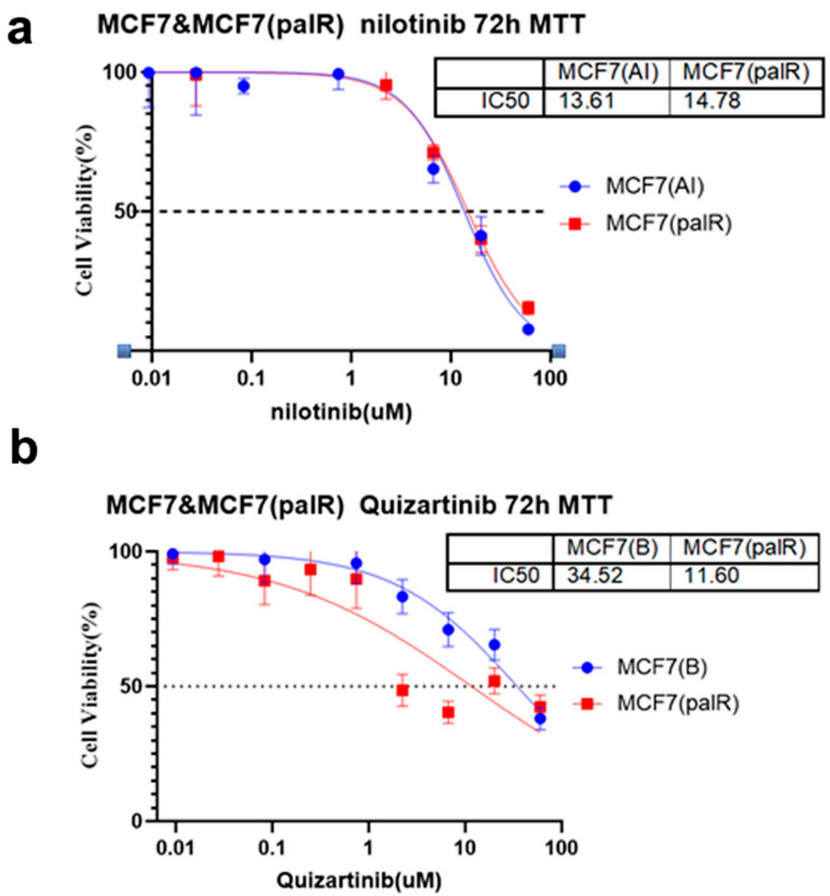

Figure S3. MCF7 drug validation results
